# Dietary Intake of Meat Cooking-Related Mutagens (HCAs) and Risk of Colorectal Adenoma and Cancer: A Systematic Review and Meta-Analysis

**DOI:** 10.3390/nu9050514

**Published:** 2017-05-18

**Authors:** Manuela Chiavarini, Gaia Bertarelli, Liliana Minelli, Roberto Fabiani

**Affiliations:** 1 Department of Experimental Medicine, University of Perugia, 06123 Perugia, Italy; manuela.chiavarini@unipg.it (M.C.); liliana.minelli@unipg.it (L.M.); 2 Department of Economics, University of Perugia, 06123 Perugia, Italy; gaiabertarelli@gmail.com; 3 Department of Chemistry, Biology and Biotechnology, University of Perugia, 06123 Perugia, Italy

**Keywords:** heterocyclic amines (HCAs), meat intake, colorectal cancer, colorectal adenomas, cancer prevention

## Abstract

Much evidence suggests that the positive association between meat intake and colorectal adenoma (CRA) and cancer (CRC) risk is mediated by mutagenic compounds generated during cooking at high temperature. A number of epidemiological studies have estimated the effect of meat-related mutagens intake on CRC/CRA risk with contradictory and sometimes inconsistent results. A literature search was carried out (PubMed, Web of Science and Scopus) to identify articles reporting the relationship between the intake of meat-related mutagens (2-amino-1-methyl-6-phenylimidazo[4,5-b]pyridine (PhIP), 2-amino-3,8-dimethylimidazo[4,5-f] quinoxaline (MeIQx), 2-amino-3,4,8-trimethylimidazo[4,5-f] quinoxaline: DiMeIQx, benzo(a) pyrene (B(a)P) and “meat derived mutagenic activity” (MDM)) and CRC/CRA risk. A random-effect model was used to calculate the risk association. Thirty-nine studies were included in the systematic review and meta-analysis. Polled CRA risk (15229 cases) was significantly increased by intake of PhIP (OR = 1.20; 95% CI: 1.13,1.28; *p* < 0.001), MeIQx (OR = 1.14; 95% CI: 1.05,1.23; *p* = 0.001), DiMeIQx (OR = 1.13; 95% CI: 1.05,1.21; *p* = 0.001), B(a)P (OR = 1.10; 95% CI: 1.02,1.19; *p* = 0.017) and MDM (OR = 1.17; 95% CI: 1.07,1.28; *p* = 0.001). A linear and curvilinear trend was observed in dose–response meta-analysis between CRA risk in association with PhIP, MDM, and MeIQx. CRC risk (21,344 cases) was increased by uptake of MeIQx (OR = 1.14; 95% CI: 1.04,1.25; *p* = 0.004), DiMeIQx (OR = 1.12; 95% CI: 1.02,1.22; *p* = 0.014) and MDM (OR = 1.12; 95% CI: 1.06,1.19; *p* < 0.001). No publication bias could be detected, whereas heterogeneity was in some cases rather high. Mutagenic compounds formed during cooking of meat at high temperature may be responsible of its carcinogenicity.

## 1. Introduction

Colorectal cancer (CRC) is the second most common cancer in women and third in men worldwide [1]. In 2012, CRC was estimated to caused 373,600 and 320,300 deaths in males and females, respectively [1]. About 55% of the CRC cases occur in more developed regions and the wide geographical variation of incidence rates across the word (10-fold) has been related to lifestyle, environmental, dietary and genetic factors. Among the dietary factors, multitude of epidemiological studies have demonstrated that meat, in particular red and processed meat, acts as risk factor for both CRC and colorectal adenoma (CRA), a benign precursor lesion of the majority of CRC [2,3,4]. Based on this evidence, the International Agency for the Research on Cancer (IARC, Lyon, France) has recently classified red meat as “probably carcinogenic to humans” (group 2A) and processed meat as “carcinogenic to humans” (group 1) [5]. 

Various mechanisms have been proposed to explain the effect of meat intake on CRC risk [6]. Meat is rich in heme iron which has been implicated in the colon carcinogenesis by promoting oxidative damage and nitrosilation [7]. In addition, meat may contain carcinogenic compounds that are generated during processing such as N-nitroso-compounds (NOCs), and cooking such as polycyclic aromatic hydrocarbons (PAHs) and heterocyclic amines (HCAs) [8]. PAHs, in particular benzo(a)pyrene (B(a)P), and HCAs are potent mutagens and carcinogens, which may explain, at least in part, the positive association between meat intake and CRC risk. 

HCAs are formed from the reaction, at high temperatures, between creatine or creatinine (found in muscle meats), amino acids, and sugars. Their formation depends upon the type of meat, the cooking method, the temperature and the duration of cooking [9,10]. HCAs are not only found in red and processed meat but also poultry and fish. Pan-frying, grilling, or barbecuing at high temperature produces the highest amounts of HCAs. Similarly, PAHs are formed during grilling or barbecuing of meat. In addition, they are also found in cured meats or smoked foods. Among the different PAHs, B(a)P is the most carcinogenic. More than 20 different HCAs have up to now been identified. The most abundant HCAs found in human diet include 2-amino-1-methyl-6-phenylimidazo[4,5-b]pyridine (PhIP), 2-amino-3,8-dimethyl imidazo [4,5-f]quinoxaline (MeIQx) and 2-amino-3,4,8-trimethylimidazo[4,5-f] quinoxaline (DiMeIQx) [9,10]. HCAs are genotoxic, mutagenic in Ames/Salmonella assays and carcinogenic in animal models. They act through the formation of DNA adducts after metabolic activation catalyzed by cytochrome P450 enzymes of the 1A family (N-oxidation) followed by O-esterification by *N*-acetyltransferases, while glucuronidation by UDP-glucuronosyltransferases (UGT) is the primary route of HCAs detoxification [9,10]. Because of the lack of human epidemiological studies, in 1993, PhIP and MeIQx were included by IARC classification in group 2B (possible human carcinogens) [11]. More recently, epidemiological investigations have evidenced the possible role of dietary HCAs exposure on the risk of human cancer in different sites such as colon, breast, prostate and pancreas [12]. Initially, the cooking methods (frying, broiling and boiling) and the degree of doneness has been employed as surrogates to estimate the dietary HCAs uptake. For this purpose, a meat cooking module within a food frequency questionnaires (FFQ) was used [13]. Subsequently, practical methods were adopted to assess the individual intake of specific HCAs in population studies by combining information on food consumption and analytical data on HCAs concentrations in specific cooked foods [14,15,16,17,18,19]. Since then, many epidemiological studies have been carried out to investigate the association between estimated HCAs intake and CRC/CRA risk with contradictory and sometime inconsistent results. 

We conducted, therefore, a systematic review of the literature to shed light on the relation between HCAs intake and CRC/CRA risk. The investigation was extended to both the “meat derived mutagenic activity” (MDM) and the B(a)P intake because in several studies on HCAs the association of CRC/CRA risk with these two parameters was also reported. In addition, we carried out for the first time, a meta-analysis to provide quantitative estimates of the association.

## 2. Materials and Methods

In this study, the standard procedures for conducting and reporting meta-analysis according to MOOSE (Meta-analysis Of Observational Studies in Epidemiology) guidelines and PRISMA (Preferred Reporting Items for Systematic reviews and Meta-Analyses) statement were followed [20,21]. 

### 2.1. Search Strategy and Data Source

A comprehensive literature search, without restrictions, until 27 January 2017 through PubMed and Web of Science was carried out to identify all the original articles on the association between HCAs intake and colorectal cancer and adenoma risk. The following search key words were used: (“heterocyclic amines” OR PhIP OR MeIQx OR DiMeIQx OR “mutagen index” OR “benzo(a)pyrene”) AND (neoplasm OR cancer OR “neoplastic disease” OR neoplasia OR tumor OR adenoma) AND (colon OR rectal OR colorectal OR intestine OR intestinal). Similarly, with the same key words, the Scopus database was also accessed to find eventually missing items. Furthermore, the reference lists of included articles and recent relevant reviews were manually examined to identify additional relevant publications.

### 2.2. Inclusion Criteria

We focused on studies in which the amount of HCAs (both as total amount and as single compounds) and B(a)P consumed by different groups was clearly reported. Potential identified articles were included if they met the following criteria: (i) used a case-control or prospective study design; (ii) evaluated the association between HCAs intake and colon, rectal and colorectal cancer (CRC) and adenomas (CRA) risk; and (iii) presented odds ratio (OR), relative risk (RR) or hazard ratio (HR) estimates with 95% confidence intervals (CIs). When there were several publications from the same study, the publication with the largest number of cases was selected. Although useful to have background information, reviews and meta-analysis were excluded. No studies were excluded for weakness of design or data quality.

### 2.3. Study Selection

After removal of duplicates, two independent investigators carried out the selection process by screening titles and abstracts for all potentially eligible studies. Disagreements between evaluators were resolved by discussion or in consultation with a third author. 

### 2.4. Data Extraction and Quality Assessment

From the selected studies we extracted the following data: the first author’s last name, year of publication, study region and design, tumor site, sample size (number of cases and controls; cohort size and incident cases), age, duration of follow-up for cohort studies, dietary assessment method and HCAs quantification method, HCAs doses comparisons, OR/RR/HR estimates with 95% confidence intervals for the highest versus lowest category of HCAs intake, matched or adjusted variables. When multiple estimates were reported in the article, we abstracted those that adjusted for the most confounding factors. The study quality was assessed by a 9-star system based on the Newcastle–Ottawa Scale method (NOS) [22]. The full score was 9 and a total score ≥7 was used to indicate high-quality study. To avoid selection bias, no study was excluded because of these quality criteria.

### 2.5. Statistical Analysis

The overall effect-size statistic estimated was the average of the logarithm of the observed odds ratio (approximated to RR when necessary) associated to the highest versus the lowest level of HCAs consumption. We used the results of the original studies from multivariable models with the most complete adjustment for potential confounders. We used random effects model and the inverse variance weighted method to calculate summary OR and 95% confidence intervals. A two-tailed *p* < 0.05 was considered statistically significant. 

We further aimed to quantify the effects of HCAs consumption on risk of CRA and CRC by a dose–response meta-analysis with random-effect models [23,24]. The linear increase in CRC/CRA risk per percentile increase in mutagens intake was estimated using the method proposed by Greenland and Longnecker, that accounts for the correlation between risk estimates for separate exposure levels depending on the same reference group, when possible [23]. For studies with non-zero or different exposure dose as reference, we adjusted the values following Liu et al. [25]. This method requires the following. (i) The number of cases and person-years (non-cases) for each level of exposure are reported; (ii) The ORs with confidence intervals are presented for at least three exposure categories; (iii) The mean or median for each category is either reported in the article or it can be estimated. When ranges of HCAs intakes were reported, the midpoint of the range was used. When the highest category was open ended, we assumed the midpoint of the category was set at the lower bounder. We used restricted cubic splines with three knots at percentiles 25%, 50%, and 75% of the distribution to evaluate the potential non-linear association between mutagen exposure and CRC/CRA. A *p*-value for curve linearity or non-linearity was calculated by testing the null hypothesis that the coefficient of the second spline is equal to zero. Linear trends with fixed effects were also tested. 

We evaluated heterogeneity between studies with Cochran’s *Q* test and used the *I*^2^ statistic to quantify the proportion of the total variation due to that heterogeneity [26]. For the *Q* statistic, a *p*-value < 0.05 was considered to be representative of statistically significant heterogeneity. The *I*^2^ statistic yields results ranged from 0 to 100% (*I*^2^ = 0–25%, no heterogeneity; *I*^2^ = 25–50%, moderate heterogeneity; *I*^2^ = 50–75%, large heterogeneity; and *I*^2^ = 75–100%, extreme heterogeneity) [27]. To explore the sources of heterogeneity among studies and test the robustness of the associations, we conducted subgroup analyses and several sensitivity analyses. We also examined the influence of individual studies on the overall risk estimate, which was investigated by recalculating the pooled estimates for the remainder of the studies by omitting one study at each turn. When in the original articles results were presented stratified by sex and gene expressions, we combined them in study’s pooled estimates, when possible. In the dose–response meta-analysis, when the resulting *I*^2^ was greater than 75% considering these adjustments we decided to consider just the original published results. 

Results of the meta-analysis may be biased if the probability of a study being published is dependent on its results. We used the methods of Begg and Mazumdar, and Egger et al. to detect publication bias [28]. Both methods test for funnel plot asymmetry, the former being based on the rank correlation between the effect estimates and their sampling variances, and the latter on a linear regression of a standard normal deviate on its precision. If a potential bias was detected, we further conducted a sensitivity analysis to assess the robustness of combined effect estimates and the possible influence of the bias and to have the bias corrected. We also conducted a sensitivity analysis to investigate the influence of a single study on the overall risk estimate by omitting one study in each turn. We considered the funnel plot to be asymmetrical if the intercept of Egger’s regression line deviated from zero with a *p*-value of less than 0.05. Subgroup analysis were conducted for the case-control and cohort studies.

The ProMeta Version 2.0 statistical program (Internovi, Via Cervese, 47522, Cesena, Italy) and packages dosresmeta 1.3.2. for R 3.1.2. (R Foundation for Statistical Computing, Vienna, Austria) was used for the analysis [29]. All reported p values are from two-sided statistical tests, and differences with *p* ≤ 0.05 were considered significant.

## 3. Results

**Figure 1:**
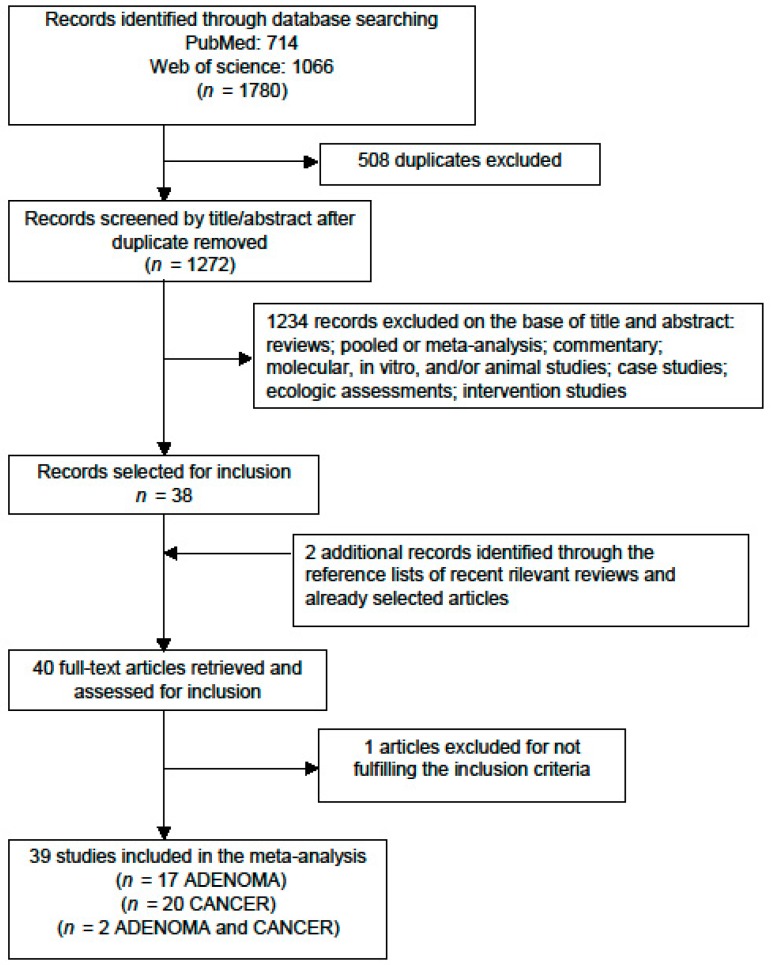
Flow diagram of systematic literature search on meat mutagen intake and colorectal adenoma (CRA) and cancer (CRC) risk.

From the primary literature research through PubMed (*n* = 714) and Web of Science (*n* = 1066) databases and after removing duplicate (*n* = 508), 1272 records were identified for title and abstract revision (Figure 1). In total, 1234 items were excluded because they were not observational epidemiological studies leaving 38 articles for full-text revision. Hand searching of reference lists of both selected articles and recent relevant reviews led to the identification of two additional papers while the search on the Scopus database did not identify further items. One article was subsequently excluded because it did not meet the inclusion criteria since it estimated the exposure to HCAs (low, intermediate and high) on the basis of red meat source and preparation but did not give their concentrations. Therefore, at the end of the selection process, 39 studies met the inclusion criteria and were enclosed in the systematic review and meta-analysis (Figure 1): 17 on CRA [30,31,32,33,34,35,36,37,38,39,40,41,42,43,44,45,46], 20 on CRC [47,48,49,50,51,52,53,54,55,56,57,58,59,60,61,62,63,64,65,66], and two studies reported data on both CRA and CRC [67,68].

### 3.1. Colorectal Adenoma (CRA)

#### 3.1.1. Study Characteristics and Quality Assessment

General characteristics of the 19 selected studies (15 case-control and four cohort) considering the association of meat mutagens with CRA are shown in Supplementary Table S1. Case-control studies were published between 2001 and 2015, 12 were population-based [31,32,35,37,39,40,42,43,44,46,67,68] and three were hospital-based [30,33,45]. Twelve were conducted in the United States [30,31,32,33,35,37,39,40,42,44,67,68] and one each in Europe [43], Canada [45] and Japan [46]. Cohort studies were published between 2006 and 2012; three were conducted in the United States [34,36,41] and one in Europe [38]. One cohort study considered the colorectal adenoma recurrence [36]. Most of the included articles used a food frequency questionnaire (FFQ) to collect dietary information. In addition, in some cases a meat module regarding the degree of doneness for various meats cooked with high-temperature methods was also used, while in other cases these parameters were included in the FFQ. In eleven studies [32,34,35,36,37,39,40,44,45,67,68] the dietary mutagenic-compounds and the meat derived mutagen index (MDM) intake were estimated using the on line Computerized Heterocyclic Amines Resource for Research in Epidemiology of Disease (CHARRED) database developed by the National Institutes of Health [69]. One study considered only the intake of B(a)P [33] while all the others reported data on both PhIP and MeIQx. The uptake of DiMeIQx was not reported in two studies [42,46] one of which reported data on MeIQ [46]. The total amount of HCAs was considered in three studies [42,46,67] while the MDM was reported in ten studies [30,32,34,35,36,37,39,40,41,44,45]. The quality score for each study (see Supplementary Tables S2 and S3, for details of quality score calculation in case-control and cohort studies, respectively) is shown in the last right column of Supplementary Table S1. The study-specific range of quality score was for from 6 to 9, the median was 7 and mean ± SD was 7.5 ± 0.8. 

#### 3.1.2. Meta-Analysis

**Figure 2:**
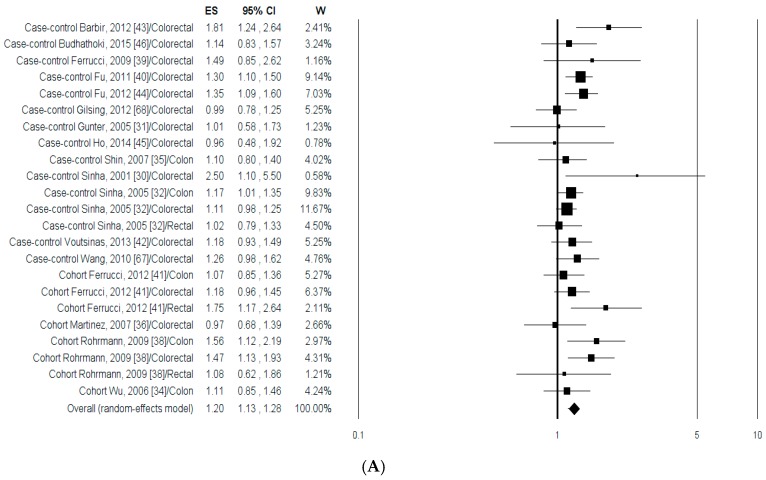

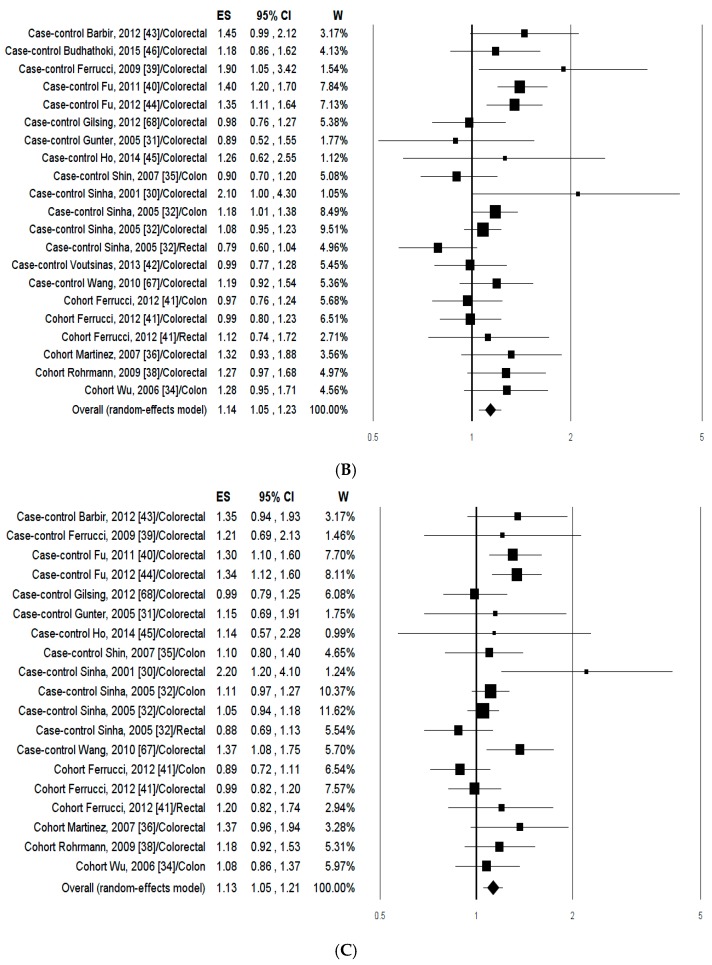

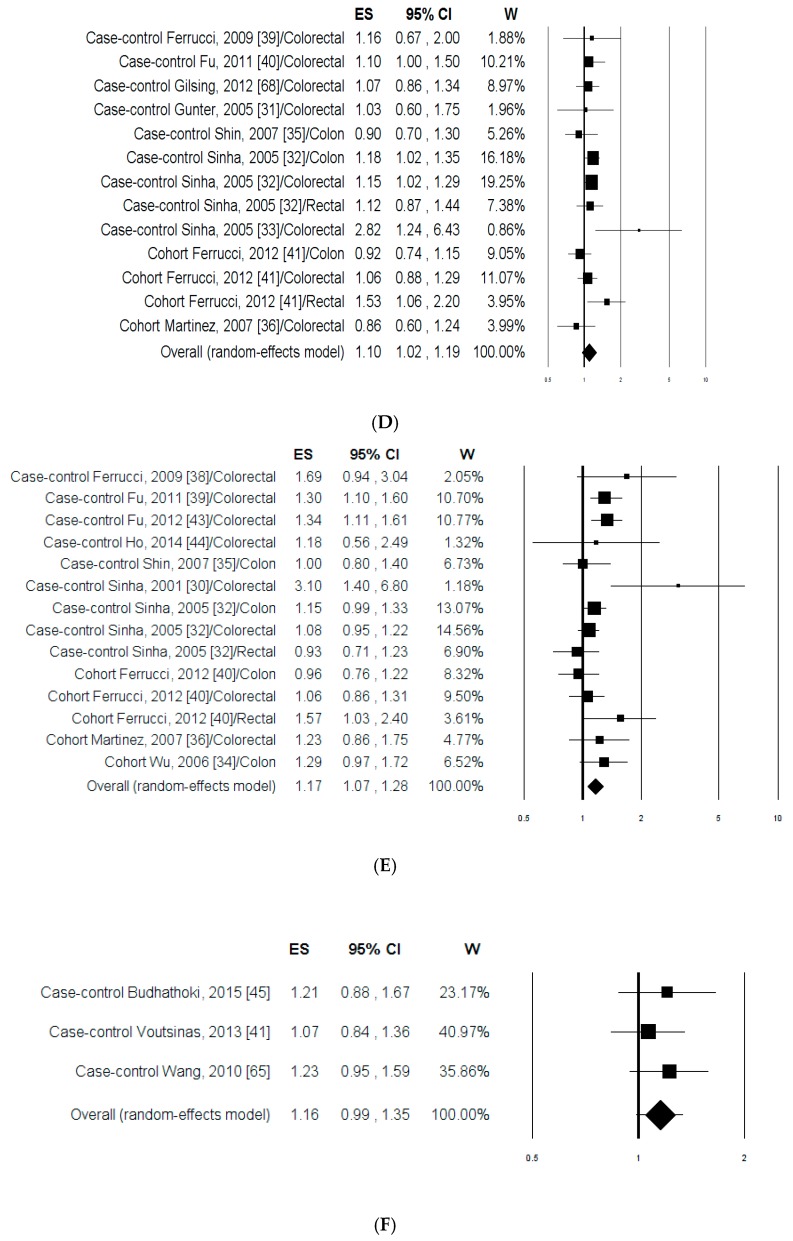
Forest plots of the highest compared with the lowest categories of intake of meat cooking-related mutagens and CRA risk: (**A**) 2-amino-1-methyl-6-phenylimidazo[4,5-b]pyridine (PhIP); (**B**) 2-amino-3,8-dimethyl imidazo [4,5-f]quinoxaline (MeIQx); (**C**) 2-amino-3,4,8-trimethylimidazo[4,5-f] quinoxaline (DiMeIQx); (**D**) Benzo(a)pyrene B(a)P; (**E**) meat-derived mutagenicity (MDM); and (**F**) total heterocyclic amines (HCAs).

The associations between CRA risk and the highest compared with the lowest intake categories of PhPI, MeIQx, DiMeIQx, B(a)P, MDM and total HCAs are shown in Figure 2. The study of Shin et al. was excluded from the calculation since the results reported the risk for a 10% increase of mutagen intake and it did not compare highest vs. the lowest values [37]. Overall, 15,229 subjects with CRA were included in the meta-analysis. For the analysis of CRA risk data from male and female [42,45,46], and from low and high risk [44] were pooled together. When data from both case-control and cohort studies were polled together the different HCAs (Figure 2A–C), B(a)P (Figure 2D) and MDM (Figure 2E) resulted significantly associated to an increment of CRA risk. On the other hand, due to the low number of studies (*n* = 3) the total HCAs uptake was not significantly associated with CRA risk (Figure 2F). 

In the pooled analysis, a moderate and statistically significant heterogeneity was observed only in the case of MeIQx (*I*^2^ = 42.37%, *p* = 0.022) (Table 1). When stratifying the analysis according to the study design, all of the parameters considered (PhIP, MeIQx, DiMeIQx, B(a)P and MDM), with the exclusion of total HCAs, resulted significantly associated to the CRA risk in the case-control studies, whereas in the cohort studies a significant effect was observed only for the PhIP (OR = 1.23; 95% CI: 1.08,1.41; *p* = 0.002) (Table 1). Heterogeneity was rather large and statistically significant in case-control studies on MeIQx (*I*^2^ = 52.52%, *p* = 0.009) and MDM (*I*^2^ = 50.28%, *p* = 0.041). Stratification of the polled data based on anatomic site showed that the highest effects were observed on colorectal adenoma. Due to the low number of data, the risk of rectal adenoma was calculated only for the exposure to PhIP. An increment of risk was observed but it was not statistically significant (Table 1). However, the PhIP was the only mutagen which significantly increased the adenoma risk in the colon (Table 1).

#### 3.1.3. Publication Bias and Sensitivity Analysis

No evidence of publication bias could be detected for risk in any case, as evidenced by both the Egger and Begg tests (Table 1), and funnel plot asymmetry (not shown). Sensitivity analyses investigating the influence of a single study on the CRA risk suggested that the estimates were not substantially modified by any single study with the exception of the effect of B(a)P which resulted reduced to not significant level (OR = 1.08; 95% CI: 0.99,1.18; *p* = 0.073) when was removed the study of Sinha et al., 2005 [32].

#### 3.1.4. Dose–Response

**Figure 3:**
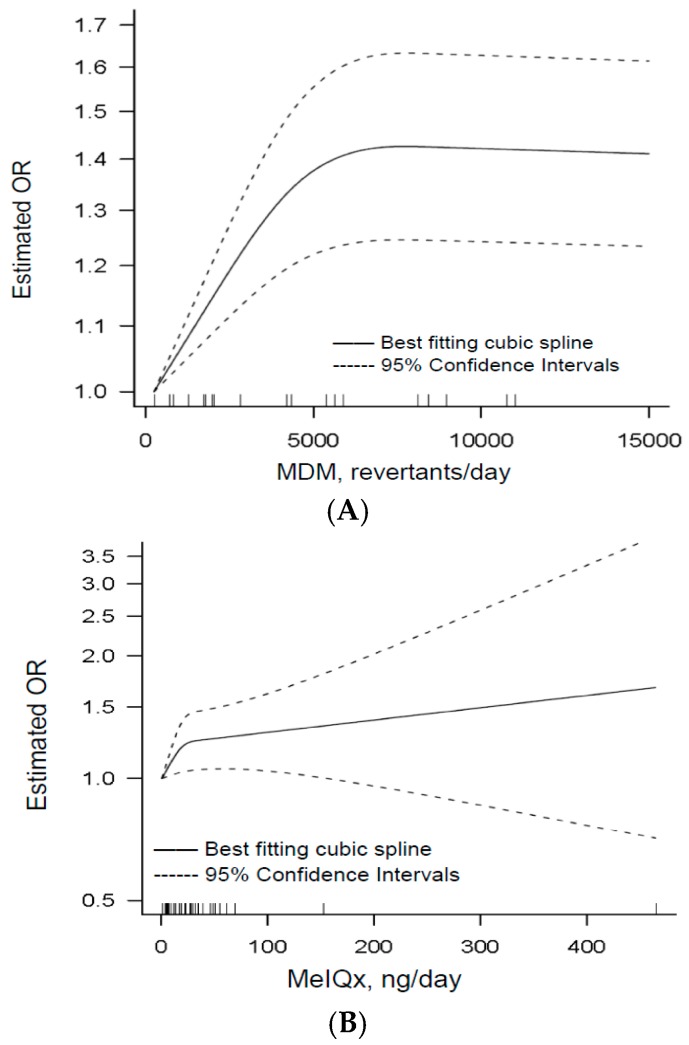

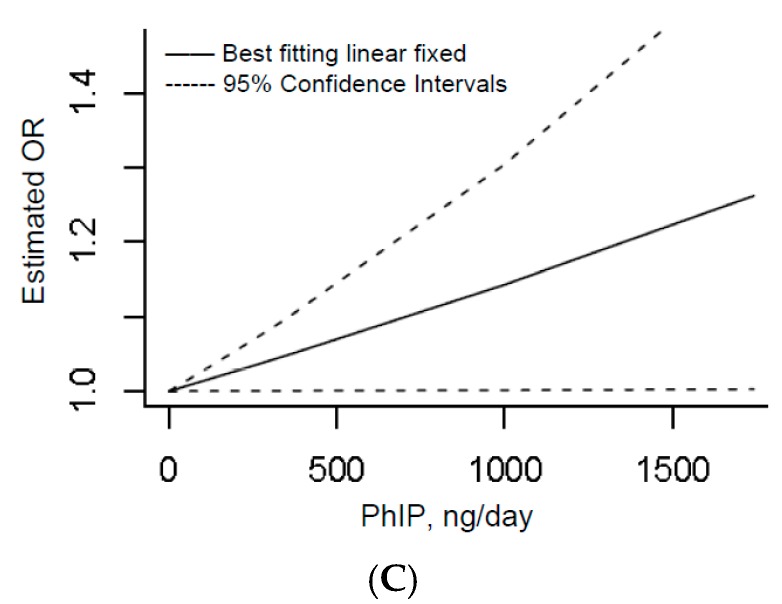
Dose-dependent plots displaying the relation between the intake of MDM (**A**), MeIQx (**B**) and PhIP (**C**), and the CRA risk.

Nine articles were identified for dose–response analysis [31,34,35,39,40,43,45,67,68]. One study was excluded because the number of cases and controls was not reported [32]. Two more studies were excluded because results were reported separately by gender and genotypes [42,46]. Using a restricted cubic splines model, we observed some evidence of a curvilinear association between MDM, MeIQx and CRA (Figure 3A,B). The dose–response meta-analysis between MDM and CRA risk was conducted on five studies [34,35,39,40,45]. A consumption of MDM corresponding to 250 revertants/day (estimated median intake in the lowest exposure category) was used as the reference to estimate all the risks. The estimated OR was 1.15 (95% CI: 1.09, 1.21; *p* < 0.0001) for 2000 revertants/day and 1.42 (95% CI: 1.24, 1.63; *p* < 0.0001) for 7000 revertants/day of MDM intake (*I*^2^ = 9%) (Figure 3A). The dose–response meta-analysis between MeIQx and CRA risk was conducted among all the above considered nine studies. Zero mg/day was the reference intake. The estimated OR was 1.26 (95% CI: 1.06, 1.49; *p* = 0.035) for 50 ng/day of MeIQx intake (*I*^2^ = 25.8%) (Figure 3B). Furthermore, PhIP intake and CRA risk was significantly positively associated in a linear fashion considering all the studies in the dose–response meta-analysis (Figure 3C). The estimated OR was 1.01 (95% CI: 1.00, 1.03; *p* = 0.04) for 100 ng/day increment of PhIP intake (*I*^2^ = 70%). There were no other statistically significant dose–response results (see Supplementary Table S4 for details).

### 3.2. Colorectal Cancer (CRC)

#### 3.2.1. Study Characteristics and Quality Assessment

General characteristics of the 22 selected studies (19 case-control and 3 cohort) considering the association of meat mutagens with CRC are shown in Supplementary Table S5. Case-control studies were published between 1997 and 2015, 16 were population-based [48,49,50,51,52,53,54,55,56,57,62,63,64,65,67,68], 2 were hospital-based [47,58] and one was cohort-based [57]. Eighteen studies were conducted in the United States [49,50,51,52,53,54,55,56,57,59,60,61,62,63,64,66,67,68] and one each in Uruguay [47], Europe [48], Japan [58] and Canada [65]. Cohort studies were published between 2010 and 2016, all of them were conducted in the United States [60,61,66]. In seven studies [60,61,62,63,65,66,68], the dietary mutagenic-compound and the mutagen index intake were estimated using the on line CHARRED database [66]. Three studies considered only the intake of MDM [49,53,55] and one study considered only the intake of MeIQx [50]. The consumption of IQ was reported in two studies [47,48], one of which reported also data on MeIQ [48]. The total amount of HCAs was considered in seven studies [48,51,58,59,61,65,67], similarly the MDM was reported in seven studies [49,52,53,55,60,62,66]. The quality score for each study (see Supplementary Tables S2 and S3, for details of quality score calculation in case-control and cohort studies, respectively) is shown in the last right column of Table S5. The study-specific range of quality score was for from 6 to 9 (median: 7 and mean ± SD: 7.5 ± 0.8).

#### 3.2.2. Meta-Analysis

**Figure 4:**
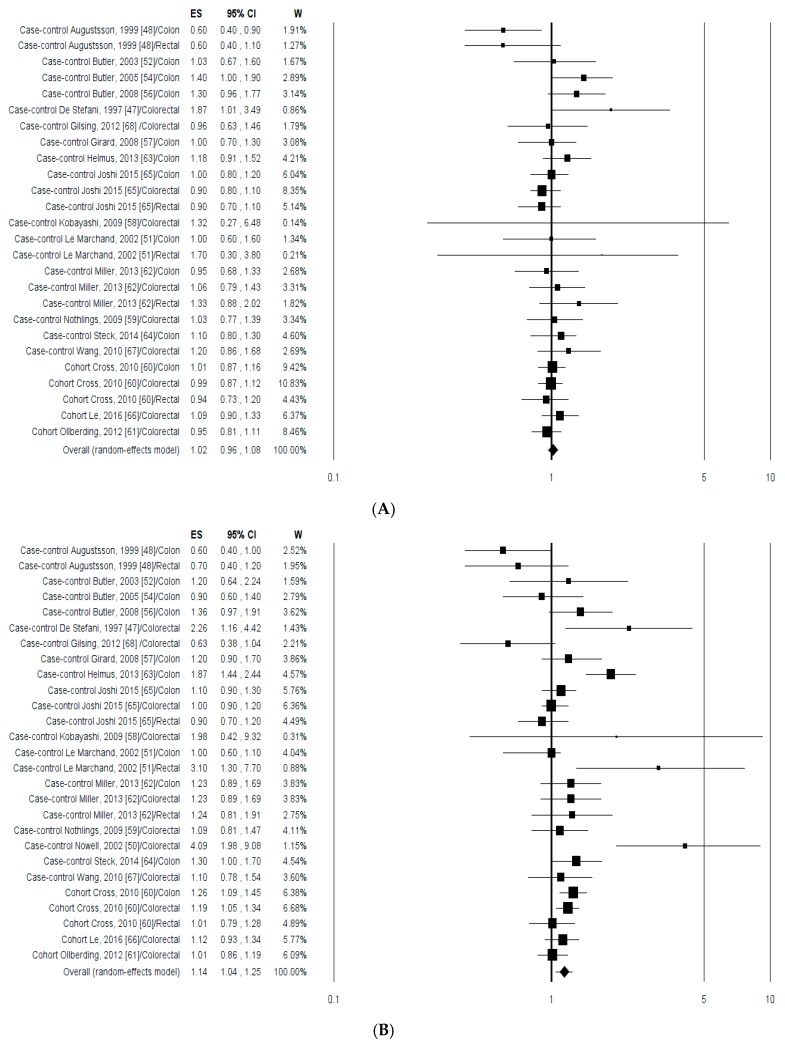

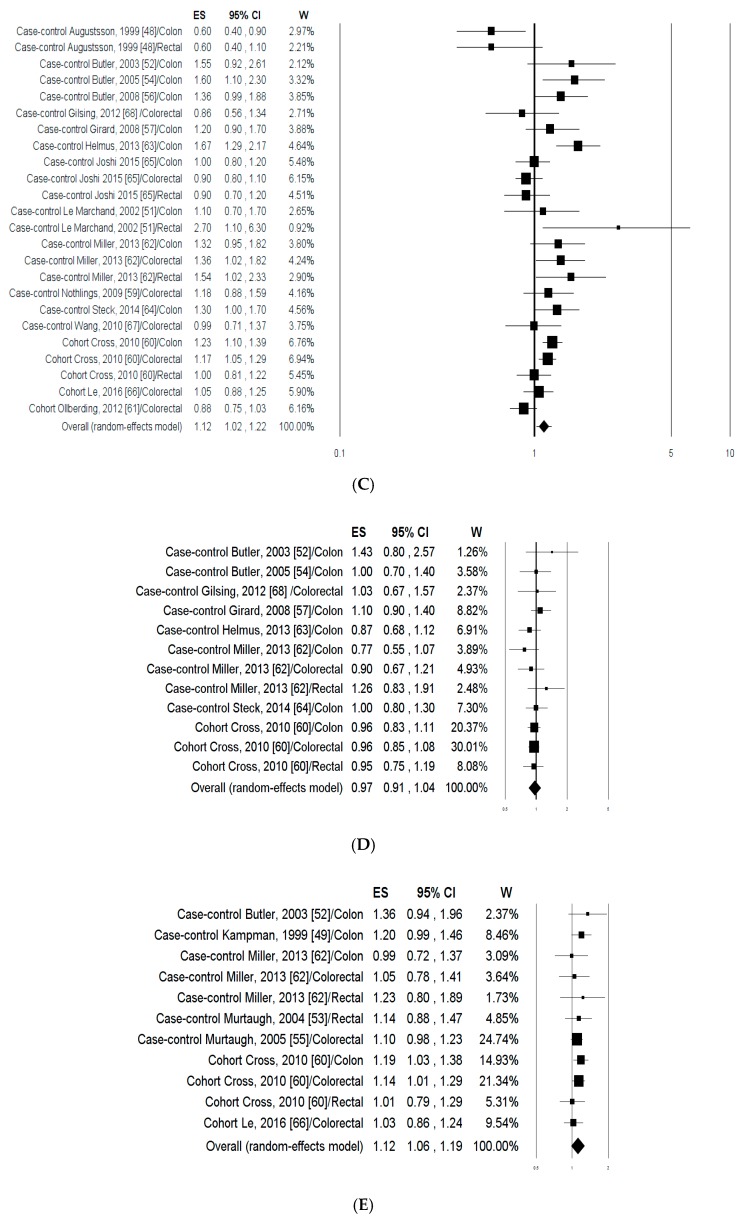

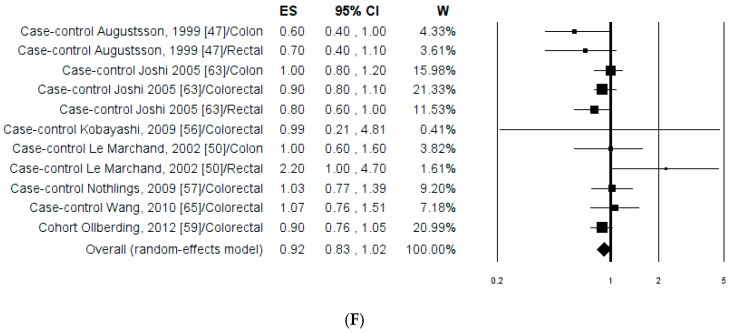
Forest plots of the highest compared with the lowest categories of intake of meat cooking-related mutagens and CRC risk: (**A**) PhIP; (**B**) MeIQx; (**C**) DiMeIQx; (**D**) B(a)P; (**E**) meat-derived mutagenicity (MDM); and (**F**) Total HCAs.

The associations between the highest compared with the lowest intake categories of PhIP, MeIQx, DiMeIQx, B(a)P, MDM and total HCAs, and the CRC risk are shown in Figure 4. For the analysis of CRC risk data from male and women [49,53,55,66], and from African Americans and Whites [52,56] were pooled together. In the case of MDM the data from red and white meat were polled together [55]. The analysis of data from both case-control and cohort studies polled together, considering overall 21344 CRC cases regardless of anatomic site, indicated that exposure to PhIP (Figure 4A), B(a)P (Figure 4D) and total HCAs (Figure 4F) was not associated to a significant variation of CRC risk. Instead, an increase of CRC risk was observed in association to the uptake of MeIQx (Figure 4B), DiMeIQx (Figure 4C) and MDM (Figure 4E). A large heterogeneity was observed for both MeIQx (*I*^2^ = 60.66%, *p* = 0.0001) and DiMeIQx (*I*^2^ = 65.88%, *p* = 0.0001) but not for MDM (*I*^2^ = 00.00%, *p* = 0.902) (Table 2). Further stratification of data analysis based on both cancer site (colon, rectal and colorectal) and study design did not reveal any statistical association of CRC risk with the intake of PhPI, B(a)P and total HCAs (Table 2). On the other hand, stratification of the polled data based on cancer site showed that exposure to MeIQx, and DiMeIQx increased the risk of colon cancer but not that of both rectal and colorectal cancer (Table 2). In addition, exposure to MDM increased the risk of both colon and colorectal cancer. Considering the study design, case-control studies showed a significant effect of exposure to MeIQx, DiMeIQx and MDM, while cohort studies produced a significant increment of CRC risk in association to MeIQx with a low heterogeneity (*I*^2^ = 27.24%, *p* = 0.240) and MDM with no heterogeneity (*I*^2^ = 0.00%, *p* = 0.527) (Table 2). 

#### 3.2.3. Publication Bias and Sensitivity Analysis

No evidence of publication bias could be detected for risk in any case as evidenced by both the Egger and Begg tests (Table 2) and funnel plot asymmetry. Sensitivity analyses investigating the influence of a single study on the CRC risk suggested that the estimates were not substantially modified by any single study.

#### 3.2.4. Dose–Response

For the dose–response analysis eight studies were considered [47,52,58,59,61,65,67,68]. We tested for linear trend with fixed and random effect and for a non-linear trend with cubic spline. No statistically significant dose–response results were obtained in any case (see Supplementary Table S4 for details).

## 4. Discussion

Many epidemiological studies have investigated the effect of meat consumption on colorectal cancer risk. The results of these studies have been revised and analyzed in several meta-analysis which have concluded that the intake of red or processed meat is associated to an increased CRA and CRC risk [2,3]. However, only more recently epidemiologist have started to consider the effects of exposure to meat-derived carcinogens such as HCAs and PAHs on CRA/CRC risk since quantifying the intake of these compounds requires more precise evaluation of meat type consumption and cooking habits. In addition, to extrapolate the mutagen doses it is necessary to use newly developed food-based mutagen database [69]. In the present investigation, we have systematically reviewed and meta-analyzed the relation between HCAs, “meat derived mutagenic activity (MDM)” and B(a)P intake and CRA/CRC risk. To the best of our knowledge, this is the first systematic review and meta-analysis that provides quantitative estimates of the association. The results of pooled analysis, regardless of study designs and anatomic neoplasia subsite, indicated that when the lowest vs. the highest intake values were compared PhIP, MeIQx, DiMeIQx, B(a)P and MDM were significantly associated with an increment of CRA risk. In the case of PhIP, MeIQx, and MDM a significant dose–response effect was also observed. On the other hand, when considering the CRC risk a significant effect was observed only associated to the intake of MeIQx, DiMeIQx and MDM. Indeed, no increment of the CRC risk was observed in association with PhIP and B(a)P exposure both in polled analysis and in stratified analysis considering separately case-control and cohort studies. In addition, no statistically significant dose–response effect was observed with any exposure in association with CRC. 

The analysis of data stratified based on neoplastic site (colon, rectal and colorectal) also showed several discrepancies between the effects exerted by the different mutagens on CRA and CRC. For instance, while PhIP, MeIQx, DiMeIQx and B(a)P significantly increased the adenoma risk in colorectal site, no effects could be observed toward carcinoma risk. These results could seem unexpected because experimental and observational studies indicate that adenoma is a valid surrogate end point for carcinoma [70]. Accordingly, it has been estimated that most of the CRC cases (70–90%) are preceded by CRA [71]. On these bases, we would expect that the mutagens that increased the risk of CRA should also increase the CRC risk. The lack of association between PhIP and B(a)P exposure with CRC risk is difficult to explain. However, it should be considered that in the studies on adenoma the control groups were recruited after colonoscopy from a population undergoing screening programs, which likely reduced the possibility of misclassification of case and control status, a condition very different from the control groups selected in the CRC studies which were recruited from the general population. It is also possible that the influence of HCAs exposure on cancer is more prominent at the early stages of the carcinogenic process. Acting as mutagenic compounds and initiators, HCAs may more deeply increase the adenoma phase and this could explain the absence of correlation with carcinoma stage. Furthermore, it is important to consider the statistic limits of meta-analysis and the very high heterogeneity evidenced in some cases. In general, a great weakness of meta-analysis is that the results used for combination are derived from studies conducted with different methods, in different populations and this may lead to high heterogeneity. In addition, pooled findings were directly driven by the included studies, which have their weaknesses relative to study design, potential bias, definition and range of mutagen intake and potential confounders by which analysis were adjusted. In particular, it is worth noting that, among the 22 studies on CRC risk included in our meta-analysis, eight did not adjust the results by smoking habit. A recent meta-analysis suggests that cigarette smoking is associated with an increased risk of CRC [72]. The ideal way to consider the smoking effect on the CRC associated to mutagen intake would be to consider separately smokers from non-smokers. Unfortunately, up to now no such data are available. In addition, it is known that the antioxidant capacity of the diet may influence the CRC risk [73]. Antioxidants are able to reduce the mutagenicity and reactivity of HCAs thus they can mask the results. However, unfortunately no study included in our meta-analysis analyzed the intake of antioxidants. Therefore, this confounding variable could not be considered in our analysis. Our data showed an higher heterogeneity in the case of CRC risk associated to MeIQx and DiMeIQx. Indeed, the heterogeneity may be the cause of the discrepancy of the results obtained between the dose–response analysis and the lowest vs. the highest intake of mutagens. In addition, this discrepancy could be due to the different number of studies used in the two analyses because some of them did not show all the information necessary for the dose–response.

Regarding the CRC risk in different anatomical subsites, we observed that in the colon the risk was increased by MeIQx, DiMeIQx and MDM while the same compounds did not affect the rectal site. This observation indicates that either the mutagens may be acting at different location within the colorectum or that various anatomical subsites have a different sensitivity toward the mutagens. Indeed, much evidence suggests that the CRC risk associated to various environmental and genetic factors, as well as the sensitivities toward chemotherapeutic drugs, is different for proximal and distal tumors [74]. Interestingly, previous data have shown that subjects who were exposed to increased levels of nitrosamines, predicted based on higher intake of red meat and processed met, were at a markedly greater increased risk for rectal cancer but not for colon cancer [75]. It can be speculated that the differences between colon and rectum responses may be due to different parameters correlated to the carcinogenesis process such as rate of xenobiotic metabolism, enzyme expression, pro-carcinogenic DNA-adducts formation, bacterial content, morphology and fecal transit time [76,77]. There is a common agreement that microbiota resident within the lumen of the gastrointestinal tract and its metabolism may influence cancer predisposition and etiology [78]. The intestinal microbiota composition may be enormously changed by the overall diet in term of probiotics and prebiotics, which may influence the results obtained. In addition, the combination of HCA and other ingredients such as fibers showed evidence that a diet high in dietary fiber sources may reduce potentially carcinogenic effects of HCA through changing their absorption and excretion [79].

The importance of the whole diet in the assessment of cancer risk is supported by several recent systematic review and meta-analysis which have considered the effects of dietary patterns instead of single components on CRC risk [80,81,82]. However, although dietary patterns representing a broader picture of food and nutrient consumption may be more predictive of disease risk, the meat type, the cooking methods and the meat doneness are further variables which vary between populations. Therefore, the meat-related mutagens exposure is likely different in various populations. Foods that contribute to HCA intake in Japan are probably different from those consumed in Western countries. Although evidence regarding meat consumption and colorectal cancer is limited in the Japanese population, a recent cohort study has showed an increment of CRC risk in man of 36% and 44% in association to the consumption of total meat and red meat, respectively [83]. Due to the low number of data (only one study carried out in Japan) we could not stratify the analysis according to the different regions, Western vs. Asian. In any case, many other factors may contributing to the risk magnitude making its assessment particularly complicated. For instance, absorption, metabolism and excretion of HCAs are further important factors in estimating the risk influencing different parameters including the production of oxygen free radicals [9]. These compounds need to be metabolically activated to exert their mutagenic activity, and the enzymes of the metabolism may have single nucleotide polymorphisms (SNP) which may influence the individual susceptibility [84]. It has been shown in some studies that the CRC risk associated to the HCAs intake is modified by N-acetyltransferase 1 (NAT1) or 2 (NAT2) genotypes [43,56]. In addition, other carcinogens and tumor promoters compounds such as heme iron and nitrosamine, may be also ingested along with HCA in food which can have an additive and/or synergistic effects [10].

## 5. Conclusions

In conclusion, this is the first meta-analysis showing that meat related mutagens may act as a risk factors of colorectal neoplasia. Our results suggest that a possible molecular mechanisms by which meat increase the colorectal cancer risk are mediated by the presence of HCAs and PAHs that are formed during cooking at high temperature. Further studies are necessary to support these findings on different populations considering more appropriately both the dose–response relationship and the effects of potential confounders such as smoking.

## Figures and Tables

**Table 1:** Results of stratified analysis of the CRA risk estimates for the highest compared with the lowest intake of PhPI, MeIQx, DiMeIQx, B(a)P, MDM and total HCAs ^1^.

	Combined Risk Estimate		Test of Heterogeneity			Publication Bias	
Mutagen	Value (95% CI)	*p*	Q	*I*^2^ %	*p*	*p* (Egger Test)	*p* (Begg Test)
**PhIP**							
Case-Control (*n* = 15) ^2^	1.19 (1.11–1.28)	<0.001	17.80	21.34	0.216	0.374	0.520
Cohort (*n* = 8 )	1.23 (1.08–1.41)	0.002	10.48	33.21	0.163	0.576	0.621
Polled (*n* = 23 )	1.20 (1.13–1.28)	<0.001	28.55	22.94	0.158	0.242	0.279
Colon (*n* = 5)	1.16 (1.05–1.29)	0.003	3.70	0.00	0.448	0.656	0.624
Rectal (*n* = 3)	1.23 (0.86–1.76)	0.248	4.91	59.23	0.086	0.695	0.602
Colorectal (*n* = 15)	1.22 (1.13–1.32)	<0.001	19.48	28.14	0.147	0.363	0.586
**MeIQx**							
Case-Control (*n* = 14)	1.14 (1.03–1.27)	0.009	29.49	52.52	0.009	0.716	0.882
Cohort (*n* = 6)	1.11 (0.99–1.25)	0.068	5.01	0.21	0.415	0.194	0.573
Polled (*n* = 21)	1.14 (1.05–1.23)	0.001	34.70	42.37	0.022	0.559	0.365
Colon (*n* = 4)	1.08 (0.93–1.26)	0.303	4.94	39.28	0.176	0.584	0.497
Rectal (*n* = 2)	–						
Colorectal (*n* = 15)	1.19 (1.09–1.30)	<0.001	21.49	34.85	0.090	0.279	0.125
**DiMeIQx**							
Case-Control (*n* = 13)	1.16 (1.06–1.27)	0.001	20.47	41.38	0.059	0.266	0.393
Cohort (*n* = 6)	1.06 (0.95–1.19)	0.308	6.15	18.73	0.292	0.068	0.091
Polled (*n* = 19)	1.13 (1.05–1.21)	0.001	28.39	36.59	0.056	0.190	0.278
Colon (*n* = 4)	1.06 (0.96–1.16)	0.275	3.04	1.39	0.385	0.634	1.000
Rectal (*n* = 2)	–						
Colorectal (*n* = 13)	1.19 (1.09–1.33)	<0.001	18.96	36.71	0.090	0.111	0.714
**B(a)P**							
Case-Control (*n* = 9)	1.13 (1.05–1.21)	0.001	7.70	0.00	0.463	0.770	1.000
Cohort (*n* = 4)	1.05 (0.86–1.28)	0.663	6.56	54.24	0.087	0.709	0.497
Polled (*n* = 13)	1.10 (1.02–1.19)	0.017	15.86	24.35	0.198	0.855	0.542
Colon (*n* = 3)	1.02 (0.84–1.24)	0.832	4.88	58.99	0.087	0.254	0.602
Rectal (*n* = 2)	–						
Colorectal (*n* = 8)	1.10 (1.01–1.20)	0.026	7.61	8.05	0.368	0.739	0.458
**MDM**							
Case-Control (*n* = 9)	1.18 (1.05–1.33)	0.005	16.09	50.28	0.041	0.256	0.297
Cohort (*n* = 5)	1.14 (0.99–1.33)	0.077	5.54	27.77	0.236	0.071	0.142
Polled (*n* = 14)	1.17 (1.07–1.28)	0.001	21.81	40.41	0.058	0.128	0.208
Colon (*n* = 4)	1.10 (0.98–1.23)	0.092	3.25	7.68	0.355	0.757	0.497
Rectal (*n* = 2)	–						
Colorectal (*n* = 8)	1.22 (1.09–1.40)	0.001	12.94	45.90	0.074	0.089	0.322
**Total HCAs**							
Case-Control (*n* = 3)							
Colorectal (*n* = 3)	1.16 (0.99–1.35)	0.063	0.70	0.00	0.706	0.607	0.602

^1^ The risk estimates ware calculated using the random-effects model; ^2^ Number of data used to calculate the risk.

**Table 2:** Results of stratified analysis of the CRC risk estimates for the highest compared with the lowest intake of PhPI, MeIQx, DiMeIQx, B(a)P, MDM and total HCAs ^1^.

	Combined Risk Estimate		Test of Heterogeneity			Publication Bias	
Mutagen	Value (95% CI)	*p*	Q	*I*^2^ %	*p*	*p* (Egger Test)	*p* (Begg Test)
**PhIP**							
Case-Control (*n* = 21) ^2^	1.03 (0.95–1.13)	0.445	29.37	31.91	0.081	0.312	0.506
Cohort (*n* = 5)	1.00 (0.93–1.07)	0.893	1.42	0.00	0.841	0.973	1.000
Polled (*n* = 26)	1.02 (0.96–1.08)	0.605	31.06	19.52	0.187	0.251	0.209
Colon (*n* = 11)	1.05 (0.95–1.16)	0.348	14.19	29.54	0.164	0.877	0.938
Rectal (*n* = 5)	0.94 (0.76–1.17)	0.593	6.68	40.13	0.154	0.719	0.624
Colorectal (*n* = 10)	1.00 (0.93–1.07)	0.951	8.25	0.00	0.509	0.055	0.128
**MeIQx**							
Case-Control (*n* = 22)	1.16 (1.02–1.32)	0.024	60.49	65.28	0.0001	0.249	0.284
Cohort (*n* = 5)	1.14 (1.04–1.24)	0.003	5.50	27.24	0.240	0.223	0.327
Polled (*n* = 27)	1.14 (1.04–1.25)	0.004	66.09	60.66	0.0001	0.471	0.428
Colon (*n* = 11)	1.18 (1.03–1.36)	0.017	24.96	59.93	0.005	0.395	0.186
Rectal (*n* = 5)	1.05 (0.80–1.38)	0.732	9.39	57.42	0.052	0.363	0.624
Colorectal (*n* = 11)	1.13 (0.99–1.29)	0.062	25.98	61.51	0.004	0.243	0.186
**DiMeIQx**							
Case-Control (*n* = 19)	1.15 (1.01–1.30)	0.036	53.82	66.56	0.0001	0.267	0.600
Cohort (*n* = 5)	1.07 (0.95–1.21)	0.255	13.56	70.50	0.009	0.165	0.327
Polled (*n* = 24)	1.12 (1.02–1.22)	0.014	67.40	65.88	0.0001	0.682	0.457
Colon (*n* = 11)	1.23 (1.07–1.41)	0.003	25.06	60.09	0.005	0.964	0.815
Rectal (*n* = 5)	1.06 (0.78–1.45)	0.693	13.67	70.74	0.008	0.534	0.624
Colorectal (*n* = 8)	1.04 (0.93–1.16)	0.534	17.20	59.31	0.016	0.726	0.805
**B(a)P**							
Case-Control (*n* = 9)	0.99 (0.89–1.09)	0.794	7.35	0.00	0.500	0.408	0.297
Cohort (*n* = 3)	0.96 (0.88–1.04)	0.334	0.01	0.00	0.997	0.206	0.117
Polled (*n* = 12)	0.97 (0.91–1.04)	0.365	7.53	0.00	0.755	0.369	0.217
Colon (*n* = 7)	0.97 (0.89–1.06)	0.530	5.63	0.00	0.465	0.698	0.881
Rectal (*n* = 2)	1.04 (0.80–1.33)	0.791	1.35	25.93	0.245	–	–
Colorectal (*n* = 3)	0.96 (0.86–1.06)	0.413	0.28	0.00	0.868	0.989	0.602
**MDM**							
Case-Control (*n* = 7)	1.13 (1.04–1.22)	0.004	2.59	0.00	0.858	0.536	0.652
Cohort (*n* = 4)	1.12 (1.03–1.21)	0.005	2.3	0.00	0.527	0.214	0.497
Polled (*n* = 11)	1.12 (1.06–1.19)	0.0001	4.83	0.00	0.902	0.997	0.697
Colon (*n* = 4)	1.18 (1.06–1.31)	0.002	1.76	0.00	0.624	0.886	0.497
Rectal (*n* = 3)	1.09 (0.93–1.29)	0.293	0.79	0.00	0.673	0.489	0.117
Colorectal (*n* = 4)	1.10 (1.02–1.18)	0.011	0.92	0.00	0.821	0.329	0.497
**Total HCAs**							
Case-Control (*n* = 10)	0.92 (0.82–1.05)	0.213	12.64	28.78	0.180	0.740	0.788
Cohort (*n* = 1)	–						
Polled (*n* = 11)	0.92 (0.83–1.02)	0.096	12.70	21.29	0.241	0.687	0.697
Colon (*n* = 3)	0.87 (0.64–1.19)	0.391	4.09	51.12	0.129	0.572	0.602
Rectal (*n* = 3)	0.97 (0.58–1.60)	0.893	6.58	69.62	0.037	0.530	0.117
Colorectal (*n* = 5)	0.93 (0.84–1.03)	0.150	1.43	0.00	0.840	0.249	0.142

^1^ The risk estimates ware calculated using the random-effects model; ^2^ Number of data used to calculate the risk.

## Supplementary Materials

The following are available online at www.mdpi.com/2072-6643/9/5/514/s1, Table S1: Characteristics of studies on HCAs, B(a)P and meat-derived mutagenic index (MDM) intake in association with colorectal adenoma (CRA) risk, Table S2: Methodological quality of case-control studies included in the meta-analysis, Table S3: Methodological quality of cohort studies included in the meta-analysis, Table S4: Statistical analysis of dose–response trend by different models on adenoma and cancer risk, Table S5: Characteristics of studies on HCAs, B(a)P and meat-derived mutagenic index intake in association with colorectal cancer (CRC) risk.
