# Supplementary material for: Dietary Intake of Meat Cooking-Related Mutagens (HCAs) and Risk of Colorectal Adenoma and Cancer: A Systematic Review and Meta-Analysis
Source: Nutrients. 2017 May 18;9(5):514. doi: 10.3390/nu9050514 (PMC5452244; doi:10.3390/nu9050514)
Supplement: Supplementary file 1 [file nutrients-09-00514-s001.zip › Supplemental TABLES.pdf]

Supplemental Table S1. Characteristics of studies on HCAs, B(a)P and meat-derived mutagenic index (MDM) intake in association with colorectal adenoma (CRA) risk.

**Author, year**<br>**Location**	**Study design,**<br>**Study name**	**Study population**<br>**Subjects (Age)**<br>**Cases/controls**<br>**Adenoma site**	**Exposure assessment**<br>**(comparisons)**	**HCAs and B(a)P doses**<br>**Meat-derived mutagenicity**	**OR/RR (95% CI)**	**Matched or adjusted variables**	**QS[1]**
Sinha, 2001 [30]<br>USA<br>Bethesda,<br>Maryland	Hospital-based<br>Case-control	146 colorectal adenoma<br>(mean:67, range:51-77y)<br>228 controls<br>(mean: 67, range:51-76y)	Self-administered FFQ[2]<br><br>Interviewer-administered meat-cooking module<br><br>(highest *vs.* lowest quintile)	PhIP[3] (ng/day)<br>0-0.94 *vs.* 140-728<br>MeIQx[4] (ng/day)<br>0-4.6 *vs.* 27-179<br>DiMeIQx[5] (ng/day)<br>Doses not reported<br>MDM[6] (Rev/day)<br>0-470 *vs.* 5661-22500	Colorectal: 2.5 (1.1-5.5)<br><br>Colorectal: 2.1 (1.0-4.3)<br><br>Colorectal: 2.2 (1.2-4.1)<br><br>Colorectal: 3.1 (1.4-6.8)	Age, sex, total caloric intake, fiber intake, reason for screening (routine or other), physical activity, smoking, use of NSAIDs[7]	7
Gunter, 2005 [31]<br>USA<br>Southern California	Population-based<br>Case-control	261 colorectal adenoma<br>(mean: 61.4±0.64y)<br>304 controls<br>(mean: 61.7±0.38y)	Self-administered FFQ<br><br>Meat preparation by in-person interview<br><br>(highest *vs.* lowest quintile)	PhIP (ng/day)<br>0-0.2 *vs.*151.8-1846.8<br>MeIQx (ng/day)<br>0-2.42 *vs.* 40.5-265.5<br>DiMeIQx (ng/day)<br>0-0.01 *vs.* 2.6-42.7<br>B(a)P[8] (ng/day)<br>0-0.29 *vs.* 31.5-515.2	Colorectal:1.01 (0.58-1.73)<br><br>Colorectal: 0.89 (0.52-1.55)<br><br>Colorectal: 1.15 (0.69-1.91)<br><br>Colorectal: 1.03 (0.6-1.75)	Age, sex, ethnicity, center, total caloric intake, fruits, vegetables saturated fat and alcoholic beverages, physical activity, smoking, NSAID, BMI[9], family history of colorectal cancer	9
Sinha, 2005 [32]<br>USA<br>Multi-center:<br>Birmingham, AL;<br>Denver, CO;<br>Detroit, MI;<br>Honolulu, HI;<br>Marshfield, WI;<br>Minneapolis, MN;<br>Pittsburgh, PA;<br>Salt Lake City, UT;<br>St. Louis, MO;<br>Washington, DC	Population-based<br>Case-control<br><br>PLCO (Prostate, Lung, Colorectal, and Ovarian Cancer Screening Trial)	2474 colon adenoma<br>688 rectal adenoma<br>3696 all adenoma<br>34817 controls<br>(mean: 62.8y)	137-items FFQ<br><br>Meat-cooking methods and doneness were included in the FFQ<br><br>Charred database[10]<br><br>(highest *vs.* lowest quintile)	PhIP (ng/day)<br>0-16.8 *vs.* 222-13334<br><br>MeIQx (ng/day)<br>0-7.0 *vs.* 46.3-1230<br><br>DiMeIQx (ng/day)<br>0-0.2 *vs.* 2.6-159<br><br>B(a)P (ng/day)<br>0-0.8 *vs.* 42.7-2168<br><br>MDM (Rev/day)<br>0-1091 *vs.* 9354-502928	Colon: 1.17 (1.01-1.35)<br>Rectal: 1.02 (0.79-1.33)<br>Colorectal: 1.11 (0.98-1.25)<br>Colon: 1.18 (1.01-1.38)<br>Rectal: 0.79 (0.60-1.04)<br>Colorectal: 1.08 (0.95-1.23)<br>Colon: 1.11 (0.97-1.27)<br>Rectal: 0.88 (0.69-1.13)<br>Colorectal: 1.05 (0.94-1.18)<br>Colon: 1.18 (1.02-1.35<br>Rectal: 1.12 (0.87-1.44)<br>Colorectal: 1.15 (1.02-1.29)<br>Colon: 1.15 (0.99-1.33)<br>Rectal: 0.93 (0.71-1.23)<br>Colorectal: 1.08 (0.95-1.22)	Age, sex, screening center, energy intake, ethnicity, educational attainment, smoking, alcohol, aspirin, ibuprofen, physical activity, BMI, folate, calcium, fiber	8
Sinha, 2005 [33]<br>USA<br>Bethesda,<br>Maryland	Hospital-based Case-control	146 colorectal adenoma<br><br>228 controls	100-items FFQ<br><br>Meat-cooking module that<br>included 23 meat items<br><br>(highest *vs.* lowest quintile)	B(a)P (ng/day)<br>(only from meat)<br>Doses not reported	Colorectal: 2.82 (1.24-6.43)	Age, sex, total caloric intake, fiber intake, reason for screening (routine or other), physical activity, smoking, use of NSAIDs	7
Wu, 2006 [34]<br>USA	Cohort study	14032 cohort (men)<br>581 distal colon	131-item FFQ<br>cooking method	PhIP (median ng/day)<br>14.4 *vs.* 220.4	Colon: 1.11 (0.85-1.46)	Age, family history of colorectal cancer, reason	9

	Health Professionals Follow-up Study (HPFS)	adenomas<br>7 years of follow-up	questionnaire<br><br>Charred database<br><br>highest *vs.* lowest quintile	MeIQx (median ng/day)<br>1.5 *vs.* 35<br>DiMeIQx (median ng/day)<br>0 *vs.* 4.0<br>MDM (median rev/day)<br>711 *vs.* 8125	Colon: 1.28 (0.95-1.71)<br><br>Colon: 1.08 (0.86-1.37)<br><br>Colon: 1.29 (0.97-1.72)	for endoscopy, negative endoscopy before 1996, physical activity, smoking, race, aspirin, total energy intake, calcium, folate	
Shin, 2007 [35]<br>USA<br>Nashville, Tennessee	Population-based Case-control<br><br>Tennessee Colorectal Polyp Study	573 adenomatous polyps (59.6±7.5 y)<br>1544 controls (57.3±7.8 y)	Telephone interview intake frequencies, and portion size of 11 meats<br><br>Charred database<br><br>highest *vs.* lowest quartile	PhIP (ng/day)<br>0-78.6 *vs.* 357.6-3128.9<br>MeIQx (ng/day)<br>0-15.3 *vs.* 84.0-846.8<br>DiMeIQx (ng/day)<br>0-1.03 *vs.* 7.05-106.3<br>B(a)P (ng/day)<br>0-9.84 *vs.* 82.67-910.7<br>MDM (rev/day)<br>0-3963 *vs.* 14184-334481	Colon: 1.1 (0.8-1.4)<br><br>Colon: 0.9 (0.7-1.2)<br><br>Colon: 1.1 (0.8-1.4)<br><br>Colon: 0.9 (0.7-1.3)<br><br>Colon: 1.0 (0.8-1.4)	Age, sex, study sites, education, indications for colonoscopy, smoking, alcohol, BMI, physical activity, NSAID, total energy intake	7
Martinez, 2007 [36]<br>USA<br>Arizona	Cohort study<br><br>Ursodeoxycholic acid (UDCA) trial	869 cohort<br>379 colorectal adenoma recurrence<br><br>31 months of follow-up	Arizona FFQ<br><br>Arizona Cancer Center Meat Preparation Questionnaire<br><br>Charred database<br><br>highest *vs.* lowest tertile	PhIP (ng/day)<br>0-27.9 *vs.* 90-1406.9<br>MeIQx (ng/day)<br>0-10.7 *vs.* 30.7-403.9<br>DiMeIQx (ng/day)<br>0-0.5 *vs.* 2.1-50.0<br>B(a)P (ng/day)<br>0-2.5 *vs.* 34.3-361.0<br>MDM (rev/day)<br>0-1710 *vs.* 4390-136556	Colorectal: 0.97 (0.68-1.39)<br><br>Colorectal: 1.32 (0.93-1.88)<br><br>Colorectal: 1.37 (0.96-1.94)<br><br>Colorectal: 0.86 (0.60-1.24)<br><br>Colorectal: 1.23 (0.86-1.75)	Age, sex, previous polyps and number of colonoscopies during follow-up	7
Shin, 2008 [37]<br>USA<br>Nashville, Tennessee	Population-based Case-control<br><br>Tennessee Colorectal Polyp Study	557 colorectal adenomatous polyps (59.6±7.5 y)<br>1493 controls (57.2±7.8 y)	Telephone interview intake frequencies, and portion size of 11 meats<br><br>Charred database<br><br>10% increment of intake	PhIP (median, ng/day)<br>Men: 193.0<br>Women: 178.4<br><br>MeIQx (median, ng/day)<br>Men: 51.9<br>Women: 29.9<br><br>DiMeIQx (median, ng/day)<br>Men: 3.5<br>Women: 2.7<br><br>B(a)P (median, ng/day)<br>Men: 37.3<br>Women: 24.9<br><br>MDM (median, rev/day)<br>Men: 8376<br>Women: 6607	Colorectal<br>Low risk: 0.99 (0.93-1.04)<br>Int. risk:1.02 (0.97-1.08)<br>High risk: 1.01 (0.91-1.12)<br>Colorectal<br>Low risk: 1.01 (0.95-1.07)<br>Int. risk: 0.98 (0.92-1.04)<br>High risk: 1.06 (0.94-1.19)<br>Colorectal<br>Low risk: 0.98 (0.93-1.04)<br>Int. risk:1.01 (0.95-1.07)<br>High risk: 1.04 (0.93-1.15)<br>Colorectal<br>Low risk: 1.02 (0.96-1.07)<br>Int. risk:0.97 (0.91-1.03)<br>High risk: 1.01 (0.91-1.12)<br>Colorectal<br>Low risk: 0.98 (0.93-1.04)<br>Int. risk: 0.99 (0.93-1.05)<br>High risk: 1.04 (0.94-1.15)	Age, sex, study site, education, indication for colonoscopy, smoking, alcohol, physical activity, and regular NSAID use	7
Rohrmann, 2009 [38]<br>Europe	Cohort study<br><br>EPIC–Heidelberg	25540 cohort<br>516 incident colorectal adenomas	FFQ<br>Questions on meat preparation methods and	PhIP (ng/day)<br><6.5 *vs.* ≥ 41.4	Colon: 1.56 (1.12-2.19)<br>Rectal: 1.08 (0.62-1.86)<br>Colorectal: 1.47 (1.13-1.93)	Age, sex, energy intake, ethanol, milk and milk product consumption,	7

Germany		(52.9±7.6 y)<br><br>3966 negative result from colonoscopy<br>(55.1±6.2 y)	preferred degree of browning<br><br>highest *vs.* lowest quartile	MeIQx (ng/day)<br><3.8 *vs.* ≥19.9<br><br>DiMeIQx (ng/day)<br>0.5 *vs.* ≥3.8	Colorectal: 1.27 (0.97-1.68)<br><br>Colorectal: 1.18 (0.92-1.53)	fiber, BMI, family history of colorectal cancer, physical activity, NSAID, smoking, education	
Ferrucci, 2009 [39]<br>USA<br>Multi-center:<br>Bethesda, Maryland;<br>Washington, D.C.;<br>San Diego, California;<br>Portsmouth, Virginia	Population-based Case-control<br><br>CONCeRN (*CO*lorectal *N*eoplasia screening with *C*olonoscopy in asymptomatic women at *R*egional *N*avy/army medical centers)	158 colorectal adenoma<br>(60.2±9.0 y)<br><br>649 controls<br>(57.2 ± 7.6 y)	124 food items DHQ[11] meat cooking module as part of the RFQ[12]<br><br>Charred database<br><br>highest *vs.* lowest quartile	PhIP (Median, ng/day)<br>4.5 (≤9) *vs.* 139.1 (>68.1)<br>MeIQx (Median, ng/day)<br>1.9 (≤3.7) *vs.* 27.9 (>18.2)<br>DiMeIQx (Median, ng/day)<br>0 (≤0.1) *vs.* 2.1 (>1.1)<br>B(a)P (Median, ng/day)<br>0.4 (≤1.6) *vs.* 47.1 (>22.2)<br>MDM (median rev/day)<br>260 (≤568) *vs.* 5654(>3162)	Colorectal: 1.49 (0.85-2.62)<br><br>Colorectal: 1.9 (1.05-3.42)<br><br>Colorectal: 1.21 (0.69-2.13)<br><br>Colorectal: 1.16 (0.67-2.00)<br><br>Colorectal: 1.69 (0.94-3.04)	Age, education, race, smoking, physical activity, BMI, study center, current HRT use, family history of colorectal polyps or cancer, NSAID, alcohol, fiber, calcium, calcium from supplements, total caloric intake	8
Wang, 2010 [67]<br>USA<br>Oahu, Hawaii	Population-based Case-control<br><br>PLCO (Prostate, Lung, Colorectal, and Ovarian Cancer Screening Trial)	914 adenoma cases<br>61 (55-68) y<br>1185 controls<br>62 (56-68) y	>200-item FFQ<br>meet module: degree of doneness for various meats cooked with high-temperature methods<br><br>highest *vs.* lowest tertile	Total HCAs (ng/day)<br><42 *vs.* >139<br>PhIP (ng/day)<br><25 *vs.* >91<br>MeIQx (ng/day)<br><8 *vs.* >40<br>DiMeIQx (ng/day)<br>0.25 *vs.* >2.0	Colorectal: 1.23 (0.95-1.59)<br><br>Colorectal: 1.26 (0.98-1.62)<br><br>Colorectal: 1.19 (0.92-1.54)<br><br>Colorectal: 1.37 (1.08-1.75)	Age, sex, ethnicity, energy intake, physical activity, recruitment site and examination procedure, BMI, smoking, alcohol, folate, aspirin, years of schooling, calcium and non-starch polysaccharides from vegetables	8
Fu, 2011 [40]*<br>USA<br>Nashville, Tennessee	Population-based case–control study<br><br>Tennessee Colorectal Polyp Study	1881 adenoma cases<br>56.8±7.7 y<br>3764 controls<br>58.5±7.3 y	Telephone interview intake frequencies, and portion size of 11 meats<br><br>Charred database<br><br>highest *vs.* lowest quartile	PhIP (ng/day)<br>≤73.3 *vs.* ≥339.4<br>MeIQx (ng/day)<br>≤12.2 *vs.* ≥70.1<br>DiMeIQx (ng/day)<br>≤0.82 *vs.* ≥5.96<br>B(a)P (ng/day)<br>≤8.93 *vs.* ≥79.9<br>MDM (rev/day)<br>≤2556 *vs.* ≥11021	Colorectal: 1.3 (1.1-1.5)<br><br>Colorectal: 1.4 (1.2-1.7)<br><br>Colorectal: 1.3 (1.1-1.6)<br><br>Colorectal: 1.1 (1.0-1.5)<br><br>Colorectal: 1.3 (1.1-1.6)	Age, sex, race, study sites, educational attainment, indications for colonoscopy, smoking, alcohol, BMI, physical activity, NSAID, total energy intake, recruitment before or after colonoscopy	7
Ferrucci, 2012 [41]<br>USA<br>Multi-center:<br>Birmingham, AL;<br>Denver, CO;<br>Detroit, MI;<br>Honolulu, HI;<br>Marshfield, WI;<br>Minneapolis, MN;<br>Pittsburgh, PA;<br>Salt Lake City, UT;	Cohort study<br><br>PLCO (Prostate, Lung, Colorectal, and Ovarian Cancer Screening Trial)	17072 cohort<br>1008 incident distal adenoma<br><br>3-5 years of follow-up	137-items FFQ<br><br>Meat-cooking methods and doneness were included in the FFQ<br><br>Charred database<br><br>highest *vs.* lowest quartile	PhIP (median, ng/day)<br>10.8 *vs.* 234.5<br><br>MeIQx (median ng/day)<br>4.8 *vs.* 62.5<br><br>DiMeIQx (median ng/day)<br>0 *vs.* 3.8<br><br>B(a)P (median ng/day)<br>0.5 *vs.* 79.0	Colon: 1.07 (0.85-1.36)<br>Rectal: 1.75 (1.17-2.64)<br>Colorectal: 1.18 (0.96-1.45)<br>Colon: 0.97 (0.76-1.24)<br>Rectal: 1.12 (0.74-1.72)<br>Colorectal: 0.99 (0.80-1.23)<br>Colon: 0.89 (0.72-1.11)<br>Rectal: 1.20 (0.82-1.74)<br>Colorectal: 0.99 (0.82-1.20)<br>Colon: 0.92 (0.74-1.15)<br>Rectal: 1.53 (1.06-2.20)	Age, sex, study centre, ethnicity, education, family history of colorectal cancer, BMI, NSAID, physical activity, smoking,<br>dietary calcium,<br>supplemental calcium,<br>dietary fibre, total energy intake	8

St. Louis, MO;<br>Washington, DC				 MDM (median rev/day)<br>692 *vs.* 9902	Colorectal: 1.06 (0.88-1.29)<br>Colon: 0.96 (0.76-1.22)<br>Rectal: 1.57 (1.03-2.40)<br>Colorectal: 1.06 (0.86-1.31)		
Gilsing, 2012 [68]<br>USA<br>Multi-center:<br>Birmingham, AL;<br>Denver, CO;<br>Detroit, MI;<br>Honolulu, HI;<br>Marshfield, WI;<br>Minneapolis, MN;<br>Pittsburgh, PA;<br>Salt Lake City, UT;<br>St. Louis, MO;<br>Washington, DC	Population-based<br>Nested Case-control<br><br>PLCO (Prostate, Lung, Colorectal, and Ovarian Cancer Screening Trial)	1205 adenoma cases<br>1386 controls	137-items FFQ with detailed meat-cooking module<br><br>Charred database<br><br>highest *vs.* lowest tertile	PhIP (ng/day)<br><42.41 *vs.* >165.0<br>MeIQx (ng/day)<br><14.99 *vs.* >49.44<br>DiMeIQx (ng/day)<br><0.57 *vs.* >2.84<br>B(a)P (ng/day)<br><2.96 *vs.* >42.85	Colorectal: 0.99 (0.78-1.25)<br><br>Colorectal: 0.98 (0.76-1.27)<br><br>Colorectal: 0.99 (0.79-1.25)<br><br>Colorectal: 1.07 (0.86-1.34)	Age, sex, ethnicity, study center, BMI, education, smoking, physical activity, total energy intake, alcohol, fiber, dietary calcium, regular use of aspirin or ibuprofen, family history of colorectal cancer	8
Voutsinas, 2013 [42]<br>USA<br>Hawaii	Population-based<br>Case-control<br><br>PLCO (Prostate, Lung, Colorectal, and Ovarian Cancer Screening Trial)	Colorectal adenoma<br>Men: 599<br>59.9 (54.2–66.0) y<br>Women: 365<br>59.8 (54.1–66.8)<br>Controls<br>Men: 800<br>59.9 (54.5–66.2) y<br>Women: 466<br>60.7 (54.8–67.5) y	268-items FFQ<br><br><br>highest *vs.* lowest tertile	Total HCAs (mean ng/day)<br><89.1 *vs.* >237<br><br>PhIP (mean ng/day)<br><49 *vs.* >140<br><br>MeIQx (mean ng/day)<br><22.4 *vs.* >82.1	Colorectal<br>Men: 1.01 (0.74-1.38)<br>Women: 1.17 (0.79-1.71)<br>Colorectal<br>Men: 1.10 (0.81-1.48)<br>Women: 1.31 (0.90-1.90)<br>Colorectal<br>Men: 0.91 (0.66-1.25)<br>Women: 1.15 (0.76-1.74)	Sex, age, race/ethnicity, initial examination modality, clinic and date of screening, family history of colorectal cancer, BMI, fibre, calcium, folate, smoking, alcohol	8
Barbir, 2012 [43]<br>Europe<br>Germany	Population-based<br>Case-control<br><br>European Prospective Investigation Cancer Nutrition<br>EPIC–Heidelberg	413 colorectal adenoma cases<br>54.5±6.20 y<br>796 controls<br>54.6±6.24 y	158-items FFQ<br><br>information on degree of browning and cooking methods<br><br>highest *vs.* lowest quartile	PhIP (ng/day)<br>0-6.72 *vs.* >42.31<br>MeIQx (ng/day)<br>0-3.77 *vs.* >18.96<br>DiMeIQx (ng/day)<br>0-0.58 *vs.* >3.7	Colorectal: 1.81 (1.24-2.64)<br><br>Colorectal: 1.45 (0.99-2.12)<br><br>Colorectal: 1.35 (0.94-1.93)	Age, sex, recruitment year, NSAIDs, family history of colorectal cancer, smoking, waist circumference, alcohol, fat	7
Fu, 2012 [44]**<br>USA<br>Nashville, Tennessee	Population-based case–control study<br><br>Tennessee Colorectal Polyp Study	1527 colorectal adenoma cases<br>56.8±7.64 y<br>3329 Controls<br>58.8±7.2 y	Telephone interview intake frequencies, and portion size of 11 meats<br><br>Charred database<br><br>highest *vs.* lowest quartile	PhIP (ng/day)<br>Doses not reported<br><br>MeIQx (ng/day)<br>Doses not reported<br><br>DiMeIQx (ng/day)<br>Doses not reported<br><br>MDM (rev/day)<br>Doses not reported	Colorectal<br>Low risk: 1.2 (0.9-1.5)<br>High risk: 1.5 (1.1-2.0)<br>Colorectal<br>Low risk: 1.2 (0.9-1.5)<br>High risk: 1.6 (1.2-2.2)<br>Colorectal<br>Low risk: 1.3 (1.0-1.6)<br>High risk: 1.4 (1.1-1.9)<br>Colorectal<br>Low risk: 1.2 (1.0-1.6)<br>High risk: 1.6 (1.2-2.2)	Age, sex, study site, educational attainment, smoking, alcohol, BMI, physical activity, NSAIDs, total energy intake, year of recruitment, and recruitment before or after colonoscopy	7
Ho, 2014 [45]	Hospital-based case–	Colorectal adenoma	Self-administered	PhIP (ng/day)	Colorectal	Sex, smoking, fruit and	6

Canada<br>Kingston, Ontario	control study	cases<br>Men: 78<br>Women: 45<br>Controls<br>Men: 65<br>Women: 148	questionnaire<br>Meat consumption questionnaire module<br><br>Charred database<br><br>highest *vs.* lowest quartile	≤116.3 *vs.* >348.92<br><br>MeIQx (ng/day)<br>≤18.54 *vs.* >55.64<br><br>DiMeIQx (ng/day)<br>≤2.06 *vs.* >6.19<br><br>MDM (rev/day)<br>≤3590 *vs.* >1069	Men: 2.14 (0.74-6.51)<br>Women: 0.56 (0.23-1.38)<br>Colorectal<br>Men: 1.67 (0.63-4.51)<br>Women: 0.94 (0.34-2.55)<br>Colorectal<br>Men: 1.98 (0.73-5.37)<br>Women: 0.68 (0.26-1.79)<br>Colorectal<br>Men: 2.58 (0.84-7.94)<br>Women: 0.63 (0.23-1.71)	vegetable intake, dietary fiber intake and biomarker levels of albumin and folate	
Budhathoki, 2015 [46]<br>Japan<br>Tokyo	Population-based case–control study<br><br>Tokyo Colorectal Adenoma Study	Adenoma cases<br>Men 498<br>61.3±5.9 y<br>Women 240<br>59.8±6.6<br>Controls<br>Men 453<br>60.0±5.7<br>Women 244<br>59.6±6.4	138-items FFQ<br><br>Meat-cooking methods and doneness were included in the FFQ<br><br>highest *vs.* lowest quartile	Total HCAs (median ng/day)<br>Men 12.3 *vs.* 53.6<br>Women 12.7 *vs.* 54.3<br>PhIP (median ng/day)<br>Men 7.4 *vs.* 33.4<br>Women 7.5 *vs.* 33.4<br>MeIQx (median ng/day)<br>Men 1.8 *vs.* 7.4<br>Women 2.2 *vs.* 7.0<br>MeIQ[13] (median ng/day)<br>Men 1.5 *vs.* 6.2<br>Women 1.6 *vs.* 6.0	Colorectal<br>Men: 1.02 (0.69-1.50)<br>Women: 1.73 (0.99-3.01)<br>Colorectal<br>Men: 1.02 (0.69-1.50)<br>Women: 1.43 (0.83-2.45)<br>Colorectal<br>Men: 1.01 (0.68-1.50)<br>Women: 1.58 (0.92-2.73)<br>Colorectal<br>Men: 0.94 (0.64-1.37)<br>Women: 2.10 (1.20-3.67)	Age, screening period, smoking, alcohol, BMI, physical activity, family history of colorectal cancer, NSAIDs. Further adjusted for age at menarche, menopausal status, and current use of hormones in females	7

[1]Quality score; [2]Food frequency questionnaire; [3]2-amino-1-methyl-6-phenylimidazo[4,5-b]pyridine; [4]2-amino-3,8-dimethyl imidazo [4,5-f]quinoxaline; [5]2-amino-3,4,8-trimethylimidazo[4,5-f] quinoxaline; [6]Meat-derived mutagenicity; [7]Nonsteroidal anti-inflammatory drugs; [8]Benzo(a)pyrene; [9]Body mass index; [10]On line database containing data on heterocyclic amines and MDM: **http://charred.cancer.gov/;** [11]Diet history questionnaire; [12]Risk factor questionnaire; [13]2-amino-3,4-dimethylimidazo[4,5-f]quinoline
*Extension of Shin, 2007 [35].**Subjects selected from the same population of Fu, 2011 [39].

Supplemental Table S2. Methodological quality of case-control studies included in the meta-analysis*

First author, publication year (reference)	Adequate definition of cases	Representativeness of cases	Selection of control subjects	Definition of control subjects	Control for important factor or additional factor†	Exposure assessment	Same method of ascertainment for all subjects	Non response Rate‡	Total quality scores
**ADENOMA**									
Sinha, 2001 [30]	✶	✶	---	✶	✶✶	✶	✶	---	**7**
Gunter, 2005 [31]	✶	✶	✶	✶	✶✶	✶	✶	✶	**9**
Sinha, 2005 [32]	✶	✶	✶	✶	✶✶	✶	✶	---	**8**
Sinha, 2005 [33]	✶	✶	---	✶	✶✶	✶	✶	---	**7**
Shin, 2007 [35]	✶	✶	✶	✶	✶✶	---	✶	---	**7**
Shin, 2008 [37]	✶	✶	✶	✶	✶✶	---	✶	---	**7**
Ferrucci, 2009 [39]	✶	✶	✶	✶	✶✶	---	✶	✶	**8**
Fu, 2011 [40]	✶	✶	✶	✶	✶✶	---	✶	---	**7**
Voutsinas, 2013 [42]	✶	✶	✶	✶	✶✶	---	✶	✶	**8**
Barbir, 2012 [43]	✶	✶	✶	✶	✶✶	---	✶	---	**7**
Fu, 2012 [44]	✶	✶	✶	✶	✶✶	---	✶	---	**7**
Ho, 2014 [45]	✶	✶	---	✶	✶✶	---	✶	---	**6**
Budhathoki, 2015 [46]	✶	✶	✶	✶	✶✶	---	✶	---	**7**
**CANCER**									
De Stefani, 1997 [47]	✶	✶	---	✶	✶	✶	✶	---	**6**
Augustsson, 1999 [48]	✶	✶	✶	✶	✶	---	✶	---	**6**
Kampman, 1999 [49]	✶	✶	✶	✶	✶✶	✶	✶	---	**8**
Nowell, 2002 [50]	✶	✶	✶	✶	✶	✶	✶	---	**7**
Le Marchand, 2002 [51]	✶	✶	✶	✶	✶✶	✶	✶	---	**8**
Butler, 2003 [52]	✶	✶	✶	✶	✶	✶	✶	---	**7**
Murtaugh, 2004 [53]	✶	✶	✶	✶	✶✶	✶	✶	✶	**9**
Butler, 2005 [54]	✶	✶	✶	✶	✶	✶	✶	---	**7**
Murtaugh, 2005 [55]	✶	✶	✶	✶	✶✶	✶	✶	✶	**9**
Butler, 2008 [56]	✶	✶	✶	✶	✶	✶	✶	---	**7**
Girard, 2008 [57]	✶	✶	✶	✶	✶	✶	✶	---	**7**
Kobayashi, 2009 [58]	✶	✶	---	✶	✶✶	---	✶	✶	**7**
Nöthlings, 2009 [59]	✶	✶	✶	✶	✶✶	---	✶	---	**7**
Miller, 2013 [62]	✶	✶	✶	✶	✶✶	✶	✶	---	**8**
Helmus, 2013 [63]	✶	✶	✶	✶	✶✶	---	✶	---	**7**
Steck, 2014 [64]	✶	✶	✶	✶	✶	✶	✶	---	**7**
Joshi, 2015 [65]	✶	✶	✶	✶	✶✶	✶	✶	---	**8**
**ADENOMA and CANCER**									
Wang, 2010 [67]	✶	✶	✶	✶	✶✶	---	✶	✶	**8**
Gilsing, 2012 [68]	✶	✶	✶	✶	✶✶	---	✶	✶	**8**

* A study could be awarded a maximum of one star for each item except for the item Control for important factor or additional factor.

† A maximum of 2 stars could be awarded for this item. Studies that controlled for age received one star, whereas studies that controlled for other important confounders (smoking and total energy intake) received an additional star.

‡ One star was assigned if there was no significant difference in the response rate between control subjects and cases by using the chi-square test (P>0.05).

Supplemental Table S3. Methodological quality of cohort studies included in the meta-analysis*

First author, publication year (reference)	Representativeness of the exposed cohort	Selection of the unexposed cohort	Ascertainment of exposure	Outcome of interest not present at start of study	Control for important factor or additional factor†	Assessment of outcome	Follow-up long enough for outcomes to occur ‡	Adequacy of follow-up of cohorts §	Total quality scores
**ADENOMA**									
Wu, 2006 [34]	✸	✸	✸	✸	✸✸	✸	✸	✸	**9**
Martinez, 2008 [36]	✸	✸	✸	✸	✸	✸	---	---	**7**
Rohrmann, 2009 [38]	✸	✸	✸	✸	✸✸	✸	---	---	**7**
Ferrucci, 2012 [41]	✸	✸	✸	✸	✸✸	✸	---	✸	**8**
**CANCER**									
Cross, 2010 [60]	✸	✸	---	✸	✸✸	✸	✸	✸	**8**
Ollberding, 2012 [61]	✸	✸	---	✸	✸✸	✸	✸	✸	**8**
Le, 2016 [66]	✸	✸	✸	✸	✸✸	✸	✸	✸	**8**

* A study could be awarded a maximum of one star for each item except for the item Control for important factor or additional factor.

† A maximum of 2 stars could be awarded for this item. Studies that controlled for age received one star, whereas studies that controlled for other important confounders (smoking and total energy intake/BMI) received an additional star.

‡ A cohort study with a follow-up time >6 y was assigned one star.

§ A cohort study with a follow-up rate >75% was assigned one star.

Supplemental Table S4. Statistical analysis of dose-response trend by different models on adenoma and cancer risk.

	Number of Studies	Model	p	I^2 %
**ADENOMA**				
**PhIP**	9	Linear Fixed	0.047	70.5
		Linear Random	0.081	70.5
		Cubic Spline	0.136	42.8
**MeIQx**	9	Linear Fixed	0.536	73.8
		Linear Random	0.060	73.8
		Cubic Spline	0.036	30.1
**DiMeIQx**	9	Linear Fixed	0.226	48.7
		Linear Random	0.066	48.7
		Cubic Spline	0.106	3.1
**B(a)P**	5	Linear Fixed	0.519	57.1
		Linear Random	0.291	57.1
		Cubic Spline	0.124	57.0
**MDM**	5	Linear Fixed	0.371	79.8
		Linear Random	0.102	79.8
		Cubic Spline	0.0001	39.0
**CANCER**				
**PhIP**	8	Linear Fixed	0.481	15.0
		Linear Random	0.481	15.0
		Cubic Spline	Not possible	
**MeIQx**	7	Linear Fixed	0.871	54.0
		Linear Random	0.894	54.0
		Cubic Spline	Not possible	
**DiMeIQx**	6	Linear Fixed	0.526	0.0
		Linear Random	0.632	0.0
		Cubic Spline	0.484	21.6
**B(a)p**	2	Linear Fixed	0.753	0.0
		Linear Random	0.753	0.0
		Cubic Spline	0.514	0.0
**HCA**	4	Linear Fixed	0.161	0.0
		Linear Random	0.161	0.0
		Cubic Spline	0.372	0.0

Supplemental Table S5. Characteristics of studies on HCAs, B(a)P and meat-derived mutagenic index intake in association with colorectal cancer (CRC) risk.

**Author, year**<br>**Location**	**Study design,**<br>**Study name**	**Study population**<br>**Subjects (Age)**<br>**Cases/controls**<br>**Tumor site**	**Exposure assessment**<br>**(comparisons)**	**HCAs and B(a)P doses**<br>**Meat-derived**<br>**mutagenicity**	**OR/RR (95% CI)**	**Matched or adjusted variables**	**QS[1]**
De Stefani, 1997 [47]<br>South America<br>Uruguay	Hospital-based<br>Case-control	250 colorectal cancer<br>500 controls	60-items FFQ[2]<br>Face to face interview<br><br>highest *vs.* lowest quartile	PhIP[3]<br>≤2.71 *vs.* >3.39<br><br>MeIQx[4]<br>≤1.27 *vs.* >1.83<br><br>IQ[5]<br>≤0.46 *vs.* >1.6	Colorectal: 1.87 (1.01-3.49)<br><br><br>Colorectal: 2.26 (1.16-4.42)<br><br><br>Colorectal:1.95 (1.03-3.68)	Age, sex, residence, education, family history of colon cancer, BMI[6], vegetable intake, total energy intake, total mean and total fat intake	6
Augustsson, 1999 [48]<br>Europe<br>Sweden	Population-based<br>Case-control	352 colon cancer<br>(mean:67, range:51-77)<br>249 rectal cancer<br>(mean:67, range:51-77)<br>553 controls<br>(mean 67, range:51-76)	188-items FFQ<br><br>postal and telephone interview<br><br>highest *vs.* lowest quintile	Total HCAs<br><br>PhIP<br><br>MeIQx<br><br>DiMeIQx[7]<br><br>IQ<br><br>MeIQ[8]	Colon: 0.6 (0.4-1.0)<br>Rectum: 0.7 (0.4-1.1)<br>Colon: 0.6 (0.4-0.9)<br>Rectum: 0.6 (0.4-1.1)<br>Colon: 0.6 (0.4-1.0)<br>Rectum: 0.7 (0.4-1.2)<br>Colon: 0.6 (0.4-0.9)<br>Rectum: 0.6 (0.4-1.1)<br>Colon: 1.1 (0.7-1.6)<br>Rectum: 0.8 (0.5-1.3)<br>Colon: 1.3 (0.9-1.8)<br>Rectum: 1.5 (1.0-2.1)	Age, sex and energy	6
Kampman, 1999 [49]<br>USA<br>California	Population-based<br>Case-control	1542 colon cancer<br>868 men, 674 women<br>1860 controls<br>989 men, 871 women	Interviewer-administered questionnaire with over 800 food items listed<br><br>highest *vs.* lowest quartile	MDM[9]	Colon<br>Men:1.3 (1.0-1.7)<br>Women: 1.1 (0.8-1.4)	Age, BMI, calories, dietary fiber, lifetime physical activity, and usual no. of cigarettes smoked	8
Nowell, 2002 [50]<br>USA<br>Arkansas, Tennessee	Population-based<br>Case-control	155 colorectal cancer<br>(mean 63.4, range: 33-87)<br>380 controls<br>(mean: 60.8, range 20-88)	In-person interviews<br><br>highest *vs.* lowest quartile	MeIQx	Colorectal: 4.09 (1.94-9.08)	Age, sex and ethnicity	7
Le Marchand, 2002 [51]<br>USA<br>Hawaii	Population-based<br>Case-control	289 colon cancer, men<br>137 rectal cancer, men<br>426 controls, men	FFQ<br>in-person interviews<br><br>highest *vs.* lowest tertile	Total HCAs<br><br>PhIP<br><br>MeIQx<br><br>DiMeIQx	Colon: 1.0 (0.6-1.6)<br>Rectum: 2.2 (1.0-4.7)<br>Colon: 1.0 (0.6-1.6)<br>Rectum: 1.7 (0.3-3.8)<br>Colon: 1.0 (0.6-1.1)<br>Rectum: 3.1 (1.3-7.7)<br>Colon: 1.1 (0.7-1.7)<br>Rectum: 2.7 (1.1-6.3)	Age, ethnicity, smoking, physical activity, aspirin use, BMI, education, non-starch polysaccharides from vegetables and total calcium	8

Butler, 2003 [52]*<br>USA<br>North Carolina	Population-based<br>Case-control	African-Americans<br>274 colon cancers<br>427 controls<br>Whites<br>346 colon cancers<br>611 controls<br>(mean 65 y)	188-items FFQ<br>in-person interviews<br><br><br>highest *vs.* lowest quintile	PhIP (median, ng/day)<br>0 *vs.* 218.5<br><br><br>MeIQx (median, ng/day)<br>4.3 *vs.* 124.2<br><br><br>DiMeIQx (median, ng/day)<br>0 *vs.* 10.3<br><br><br>B(a)P[10] (median, ng/day)<br>0.5 *vs.* 78.2<br><br><br>MDM (median, rev/day)<br>800 *vs.* 17600	African-Americans<br>Colon: 0.9 (0.4-1.9)<br>Whites<br>Colon: 1.1 (0.7-2.0)<br>African-Americans<br>Colon: 1.2 (0.5-3.2)<br>Whites<br>Colon: 1.2 (0.5-2.7)<br>African-Americans<br>Colon: 2.1 (0.9-5.1)<br>Whites<br>Colon: 1.3 (0.7-2.6)<br>African-Americans<br>Colon: 2.0 (1.1-3.6)<br>White<br>Colon: 1.1 (0.7-1.7)<br>African-Americans<br>Colon: 1.7 (0.9-3.1)<br>Whites<br>Colon: 1.2 (0.8-2.0)	Age, race, sex, energy-adjusted fat intake, energy intake, fiber intake, and offsets.	7
Murtaugh, 2004 [53]<br>USA<br>California, Utah	Population-based<br>Case-control	559 rectal cancers, men<br>672 controls, men<br>393 rectal cancers, women<br>532 controls, women	Recall diet history<br>in-person interviews	MDM (median, rev/day)<br><351 *vs.* >988 (men)<br><390 *vs.* >1092 (women)	Rectum<br>Men: 1.37 (0.98-1.92)<br>Women: 0.89 (0.60-1.31)	Age, BMI, energy intake, dietary fiber, calcium, lifetime physical activity, and usual number of cigarettes smoked	9
Butler, 2005 [54]*<br>USA<br>North Carolina	Population-based<br>Case-control	400 colon cancers<br>412 controls	150-items FFQ<br>in-person interviews<br><br><br>Exposed group *vs.* reference group (individuals with less than the median dietary intake)	PhIP (median, ng/day)<br><39.1 *vs.* ≥39.1<br>MeIQx (median, ng/day)<br><36.7 *vs.* ≥36.7<br>DiMeIQx (median, ng/day)<br><2.2 *vs.* ≥2.2<br>B(a)P (median, ng/day)<br><6.4 *vs.* ≥6.4	Colon: 1.4 (1.0-1.9)<br><br>Colon: 0.9 (0.6-1.4)<br><br>Colon: 1.6 (1.1-2.3)<br><br>Colon: 1.0 (0.7-1.4)	Age, race, sex, total meat, energy-adjusted fat intake, dietary fiber intake, total energy, and offsets	7
Murtaugh, 2005 [55]<br>USA<br>California, Utah	Population-based<br>Case-control	1315 colorectal cancers, men<br>1518 controls, men<br>983 colorectal cancers, women<br>1231 controls, women	Recall diet history<br>in-person interviews	MDM (median, rev/day)<br>Red meat:<br>≤156 *vs.* >416 (men);<br>≤156 *vs.* >416 (women)<br>White meat:<br>≤156 *vs.* >351 (men)<br>≤208 *vs.* >468 (women)	 Colorectal - Red meat<br>Men: 1.05 (0.86-1.28)<br>Women:0.96 (0.67-1.38)<br>Colorectal - White meat<br>Men: 1.31 (1.08-1.58)<br>Women: 0.94 (0.74-1.19)	Age, BMI, energy intake, dietary fiber, calcium, lifetime physical activity, and usual number of cigarettes smoked	9

Butler, 2008 [56]*<br>USA<br>North Carolina	Population-based<br>Case-control	African-American<br>217 colon cancers<br>315 controls<br>White<br>290 colon cancers<br>534 controls	150-items FFQ<br>in-person interviews<br><br>highest *vs.* lowest tertile	PhIP (median, ng/day)<br>3.7 *vs.* 148.2<br><br><br>MeIQx (median, ng/day)<br>8.0 *vs.* 90.8<br><br><br>DiMeIQx (median, ng/day)<br>0.3 *vs.* 7.6	African Americans<br>Colon: 1.3 (0.8-2.1)<br>White<br>Colon: 1.3 (0.9-2.0)<br>African Americans<br>Colon: 1.3 (0.8-2.3)<br>White<br>Colon: 1.4 (0.9-2.2)<br>African Americans<br>Colon: 1.7 (1.0-2.9)<br>White<br>Colon: 1.2 (0.8-1.8)	Age, race, sex, energy-adjusted fat intake, dietary fiber intake, total energy, offsets, and individual heterocyclic amines	7
Girard, 2008 [57]*<br>USA<br>North Carolina	Population-based<br>Case-control	537 colon cancers<br>866 controls	150-items FFQ<br>in-person interviews<br><br>High exposed group *vs.* low exposed group (variable cut points are median values based on the distribution among controls)	PhIP (median, ng/day)<br><45.9 *vs.* ≥45.9<br>MeIQx (median, ng/day)<br><37.3 *vs.* ≥37.3<br>DiMeIQx (median, ng/day)<br><2.4 *vs.* ≥2.4<br>B(a)P (median, ng/day)<br><7.7 *vs.* ≥7.7	Colon: 1.0 (0.7-1.3)<br><br>Colon: 1.2 (0.9-1.7)<br><br>Colon: 1.2 (0.9-1.7)<br><br>Colon: 1.1 (0.9-1.4)	Age, race, sex, energy-adjusted fat intake, dietary fiber intake, total energy, offsets, and all heterocyclic amines	7
Kobayashi, 2009 [58]<br>Japan<br>Nagano Prefecture	Hospital-based Case-control	117 colorectal cancer<br>(21-76 y, mean 59.7 y)<br>238 controls<br>(mean 59.6 y)	144-items FFQ<br>self-administered<br><br><br>highest *vs.* lowest tertile	Total HCAs (mean ng/day)<br>21.7 *vs.* 123.8<br>PhIP (mean ng/day)<br>13.2 *vs.* 76.0<br>MeIQ (mean ng/day)<br>2.8 *vs.* 13.8<br>MeIQx (mean ng/day)<br>3.0 *vs.* 15.7	Colorectal: 0.99 (0.21-4.81)<br><br>Colorectal: 1.32 (0.27-6.48)<br><br>Colorectal:1.23 (0.23-6.64)<br><br>Colorectal: 1.98 (0.42-9.32)	Smoking status, alcohol intake family history of colorectal cancer, body mass index, JA membership, and intake of vegetables, meat, fish, and dietary fiber	7
Nöthlings, 2009 [59]<br>USA<br>Hawaii–Los Angeles	Cohort-based Case-control<br><br>Hawaii–Los Angeles Multiethnic Cohort Study	398 colorectal cancer<br>(62-74 y)<br>1444 controls<br>(60-72)	>180-items FFQ<br>cooked meat module<br>self-administered<br><br><br><br>highest *vs.* lowest tertile	Total HCAs (ng/1000 kcal/day)<br>0-217.3 *vs.* >566.9<br>PhIP (ng/1000 kcal/day)<br>0-171.7 *vs.* >460.5<br>MeIQx (ng/1000 kcal/day)<br>0-29.8 *vs.* >93.5<br>DiMeIQx (ng/1000 kcal/day)<br>0-1.8 *vs.* >6.2	Colorectal: 1.03 (0.77-1.39)<br><br>Colorectal: 1.03 (0.77-1.39)<br><br>Colorectal: 1.09 (0.81-1.47)<br><br>Colorectal: 1.18 (0.88-1.59)	Sex, age, ethnicity, family history of colorectal cancer, BMI, dietary fiber, calcium, vitamin D, folic acid, ethanol, meat, physical activity, smoking	8

Cross, 2010 [60]<br>USA<br>California, Florida, Louisiana, New Jersey, North Carolina, Pennsylvania.<br>Atlanta and Detroit	Cohort study<br>NIH-AARP Diet and Health Study	300,948 participants<br>175,369 men<br>125,579 women<br>50-71 y<br>7.2 years of follow-up<br>1,995 colon cancers<br>724 rectal cancers	124-item FFQ<br>meat cooking module as part of the RFQ[11]<br>self-administered<br><br>Charred database[12]<br><br>highest *vs.* lowest quintile	PhIP (median, ng/1000 kcal/day)<br>2.1 *vs.* 123.6<br>MeIQx (median, ng/1000 kcal/day)<br>0.5 *vs.* 24.4<br>DiMeIQx (median, ng/1000 kcal/day)<br>0 *vs.* 1.74<br>B(a)P (median, ng/1000 kcal/day)<br>0.21 *vs.* 43.97<br>MDM (median, rev/1000 kcal/day)<br>165 *vs.* 4349	Colon: 1.01 (0.87-1.16)<br>Rectum: 0.94 (0.73-1.20)<br>Colorectal: 0.99 (0.87-1.12)<br>Colon: 1.26 (1.09-1.45)<br>Rectum: 1.01 (0.79-1.28)<br>Colorectal: 1.19 (1.05-1.34)<br>Colon: 1.23 (1.10-1.39)<br>Rectum: 1.00 (0.81-1.22)<br>Colorectal: 1.17 (1.05-1.29)<br>Colon: 0.96 (0.83-1.11)<br>Rectum: 0.95 (0.75-1.19)<br>Colorectal: 0.96 (0.85-1.08)<br>Colon: 1.19 (1.03-1.38)<br>Rectum: 1.01 (0.79-1.29)<br>Colorectal: 1.14 (1.01-1.29)	Sex, education, BMI, smoking, total energy, fiber, and calcium	8
Wang, 2010 [67]<br>USA<br>Oahu	Population-based Case-control<br><br>Hawaii Surveillance, Epidemiology and End Results cancer registry Controls	496 colorectal cancer<br>Median 66 y<br>607 controls<br>Median 67 y	>200-item FFQ<br>meet module: degree of doneness for various meats cooked with high-temperature methods<br><br>highest *vs.* lowest tertile	Total HCAs (mean ng/day)<br><42 *vs.* >139<br>PhIP (mean ng/day)<br><25 *vs.* >91<br>MeIQx (mean ng/day)<br><8 *vs.* >40<br>DiMeIQx (mean ng/day)<br>0.25 *vs.* >2.0	Colorectal: 1.07 (0.76-1.51)<br><br>Colorectal: 1.20 (0.86-1.68)<br><br>Colorectal: 1.10 (0.78-1.54)<br><br>Colorectal: 0.99 (0.71-1.37)	Age, sex, ethnicity, energy intake, physical activity, recruitment site and examination procedure, BMI, smoking, alcohol, folate, aspirin, years of schooling, calcium and non-starch polysaccharides from vegetables	8
Ollberding, 2012 [61]<br>USA<br>California, Hawaii	Cohort study<br>Multiethnic Cohort (MEC) Study	131,763 participants<br>45-75 y<br>8.1 years of follow up<br>1,757 colorectal cancer	Quantitative-FFQ<br>meat-cooking module<br><br>Charred database<br><br>highest *vs.* lowest quintile	Total HCAs (median, ng/1000 kcal/day)<br>43.82 *vs.* 1237.86<br>PhIP (median, ng/1000 kcal/day)<br>35.34 *vs.* 1027.3<br>MeIQx (median, ng/1000 kcal/day)<br>3.09 *vs.* 208.18<br>DiMeIQx (median, ng/1000 kcal/day)<br>0.15 *vs.* 16.75	Colorectal: 0.90 (0.76-1.05)<br><br>Colorectal: 0.95 (0.81-1.11)<br><br>Colorectal: 1.01 (0.86-1.19)<br><br>Colorectal: 0.88 (0.75-1.03)	Age, sex, ethnicity, family history of colorectal cancer and polyp, BMI, smoking, nonsteroidal anti-inflammatory use, alcohol, physical activity, history of diabetes, hormone replacement therapy (females only), total calories, dietary fiber, calcium, folate, and vitamin D	8
Gilsing, 2012 [68]<br>USA<br>Multi-center:<br>Birmingham, AL;<br>Denver, CO;<br>Detroit, MI;<br>Honolulu, HI;	Population-based Nested Case-control<br><br>PLCO (Prostate, Lung, Colorectal, and Ovarian Cancer Screening Trial)	364 colorectal cases<br>394 controls	137-items FFQ<br>with detailed meat-cooking module<br><br>Charred database<br><br>highest *vs.* lowest tertile	PhIP (ng/day)<br><42.41 *vs.* >165.0<br>MeIQx (ng/day)<br><14.99 *vs.* >49.44<br>DiMeIQx (ng/day)<br><0.57 *vs.* >2.84<br>B(a)P (ng/day)	Colorectal: 0.96 (0.63-1.46)<br><br>Colorectal: 0.63 (0.38-1.04)<br><br>Colorectal: 0.86 (0.56-1.34)<br><br>Colorectal: 1.03 (0.67-1.57)	Age, sex, ethnicity, study center, BMI, education, smoking, physical activity, total energy intake, alcohol, fiber, dietary calcium, regular use of aspirin or	8

Marshfield, WI;<br>Minneapolis, MN;<br>Pittsburgh, PA;<br>Salt Lake City, UT; St. Louis, MO;<br>Washington, DC				<2.96 *vs.* >42.85		ibuprofen, family history of colorectal cancer	
Miller, 2013 [62]<br>USA<br>Central and northeast Pennsylvania	Population-based<br>Case-control	989 colorectal cases<br>693 colon<br>289 rectal<br>mean 61.4 y<br><br>1033 controls<br>mean 66.5 y	137 items FFQ- DHQ[13]<br>in-person interviews<br><br>Charred database<br><br>highest *vs.* lowest quintile	PhIP (median, ng/1000 kcal/day)<br><7.2 *vs.* >68.3<br>MeIQx (median, ng/1000 kcal/day)<br><4.2 *vs.* >23.8<br>DiMeIQx (median, ng/1000 kcal/day)<br><0.23 *vs.* >2.2<br>B(a)P (median, ng/1000 kcal/day)<br><0.32 *vs.* >19.0<br>MDM (median, revertants/ 1000kcal/day)<br><665 *vs.* >3995	Colon: 0.95 (0.68-1.33)<br>Rectum: 1.33 (0.88-2.02)<br>Colorectal: 1.06 (0.79-1.43)<br>Colon: 1.23 (0.89-1.69)<br>Rectum: 1.24 (0.81-1.91)<br>Colorectal: 1.22 (0.91-1.64)<br>Colon: 1.32 (0.95-1.82)<br>Rectum: 1.54 (1.02-2.33)<br>Colorectal: 1.36 (1.02-1.82)<br>Colon: 0.77 (0.55-1.07)<br>Rectum: 1.26 (0.83-1.91)<br>Colorectal: 0.90 (0.67-1.21)<br>Colon: 0.99 (0.72-1.37)<br>Rectum: 1.23 (0.80-1.89)<br>Colorectal: 1.05 (0.78-1.41)	Age, sex, total energy intake, smoking, BMI, past regular NSAID[14] use, fruit and vegetable consumption	8
Helmus, 2013 [63]<br>USA<br>Kentucky	Population-based<br>Case-control	1062 colon cancer cases<br>mean 62.6 y<br>1645 controls<br>mean 61.4 y	175 items FFQ<br>Meat Preparation Questionnaire<br>self-administered<br><br>Charred database<br><br>highest *vs.* lowest quartile	PhIP<br>Doses not reported<br>MeIQx<br>Doses not reported<br>DiMeIQx<br>Doses not reported<br>B(a)P<br>Doses not reported	Colon: 1.18 (0.91-1.52)<br><br>Colon: 1.87 (1.44-2.44)<br><br>Colon: 1.67 (1.29-2.17)<br><br>Colon: 0.87 (0.68-1.12)	Age, sex, race, waist-hip ratio, average daily total caloric intake, family history of colorectal cancer, smoking, NSAID use	7
Steck, 2014 [64]*<br>USA<br>North Carolina	Population-based<br>Case-control	536 colon cancer cases<br>862 controls<br>mean 65 y	150-items FFQ<br>in-person interviews<br><br>High exposed group *vs.* low exposed group (variable cut points are median values based on the distribution among controls)	PhIP (ng/day)<br><45.9 *vs.* ≥45.9<br>MeIQx (ng/day)<br><37.3 *vs.* ≥37.3<br>DiMeIQx (ng/day)<br><2.4 *vs.* ≥2.4<br>B(a)P (ng/day)<br><7.7 *vs.* ≥7.7	Colon: 1.1 (0.8-1.3)<br><br>Colon: 1.3 (1.0-1.7)<br><br>Colon: 1.3 (1.0-1.7)<br><br>Colon: 1.0 (0.8-1.3)	Age, sex, race, offsets, total energy intake, energy-adjusted fat intake, dietary fiber intake, and other HCA variables	7
Joshi, 2015 [65]<br>Canada-USA<br><br>Ontario, Hawaii, Southern California Consortium	Population-based<br>Case-control	3350 colorectal cases<br>mean 59.4 y<br>3504 controls<br>mean 57.8 y	>200-item FFQ<br>meat cooking module<br><br>Face-to-face interview, Computer-Assisted Telephone Interview (CATI), or mail for self-administration	Total HCAs (ng/1000 kcal/day)<br>0-82.04 *vs.* 387.97-6166.49<br><br>PhIP (ng/1000 kcal/day)<br>0-60.61 *vs.* 315.12-	Colon: 1.0 (0.8-1.2)<br>Rectum: 0.8 (0.6-1.0)<br>Colorectal: 0.9 (0.8-1.1)<br><br>Colon: 1.0 (0.8-1.2)<br>Rectum: 0.9 (0.7-1.1)<br>Colorectal: 0.9 (0.8-1.1)	Age, sex, BMI, race, saturated fat, dietary fiber, center, vegetables, physical activity, total calorie intake, smoking	8

			Charred database<br><br>highest *vs.* lowest quintile	4922.97<br><br>MeIQx (ng/1000 kcal/day)<br>0-11.83 *vs.* 70.03-1188.52<br><br>DiMeIQx (ng/1000 kcal/day)<br>0-0.82 *vs.* 4.71-173.75	Colon: 1.1 (0.9-1.3)<br>Rectum: 0.9 (0.7-1.2)<br>Colorectal: 1.0 (0.9-1.2)<br><br>Colon: 1.0 (0.8-1.2)<br>Rectum: 0.9 (0.7-1.2)<br>Colorectal: 0.9 (0.8-1.1)		
Le, 2016 [66]<br>USA	Cohort study<br>2 cohorts:<br>1. Health Professionals Follow-up Study (HPFS)<br><br>2. Nurses' Health Study (NHS)	29,615 men<br>40-75 y<br>418 colorectal cancer<br><br>65,785 women<br>30-55 y<br>790 colorectal cancer<br><br>14 years of follow up	Validate 131-item FFQ cooking questionnaire<br><br>Charred database<br><br>highest *vs.* lowest quintile	PhIP<br>M: 0-33.5 *vs.*165.4-2495.6<br>F: 0-35.6 *vs.*229.9-1665.7<br><br>MeIQx<br>M: 0-4.2 *vs.* 63.5-353.2<br>F: 0-5.7 *vs.* 143.8-970.2<br><br>DiMeIQx<br>M: 0-0 *vs.* 1.9-39<br>F: 0-0.6 *vs.* 17.4-120.0<br><br>MDM<br>M: 0-1755 *vs.* 7717-54285<br>F: 0-2408 vs. 12981-171620	Colorectal<br>Male: 1.01 (0.72-1.41)<br>Female: 1.14 (0.90-1.45)<br><br>Colorectal<br>Male: 1.22 (0.89-1.68)<br>Female: 1.07 (0.86-1.34)<br><br>Colorectal<br>Male: 0.88 (0.65-1.19)<br>Female: 1.15 (0.93-1.43)<br><br>Colorectal<br>Male: 1.02 (0.73-1.41)<br>Female: 1.04 (0.83-1.30)	Age, 2-year follow-up cycle, family history of colorectal cancer in first degree relatives, prior lower gastrointestinal endoscopy, smoking, BMI, physical activity, aspirin or NSAID, total caloric intake, alcohol	9

[1]Quality score; [2]Food frequency questionnaire; [3]2-amino-1-methyl-6-phenylimidazo[4,5-b]pyridine; [4]2-amino-3,8-dimethyl imidazo [4,5-f]quinoxaline; [5]2-amino-3-methylimidazo[4,5-*f*] quinoline; [6]Body mass index; [7]2-amino-3,4,8-trimethylimidazo[4,5-f] quinoxaline; [8]2-amino-3,4-dimethylimidazo[4,5-f]quinoline; [9]Meat-derived mutagenicity; [10]Benzo(a)pyrene; [11]Risk factor questionnaire;[12]On line database containing data on heterocyclic amines and MDM: **http://charred.cancer.gov/;** [13]Diet history questionnaire; [14]Nonsteroidal anti-inflammatory drugs;
*data from these studies were derived from the same recruited population.
